# Transcriptome Inference and Systematic Approaches to Investigate TCM Compounds With Beneficial Metabolic Effects

**DOI:** 10.1002/ptr.70407

**Published:** 2026-07-15

**Authors:** Jing Li, Yang‐Chi‐Dung Lin, Hsi‐Yuan Huang, Jihang Chen, Huali Zuo, Yun Tang, Hsien‐Da Huang

**Affiliations:** ^1^ School of Medicine, the Chinese University of Hong Kong Shenzhen Guangdong PR China; ^2^ Warshel Institute for Computational Biology, School of Medicine, the Chinese University of Hong Kong Shenzhen Guangdong PR China; ^3^ Guangdong Provincial Key Laboratory of Digital Biology and Drug Development The Chinese University of Hong Kong Shenzhen Guangdong PR China; ^4^ Institute of Bioinformatics and Systems Biology, College of Engineering Bioscience, National Yang Ming Chiao Tung University Hsinchu Taiwan

**Keywords:** CMap analysis, molecular docking, network pharmacology, obesity, transcriptomic analysis, type 2 diabetes mellitus

## Abstract

Obesity is a major risk factor for type 2 diabetes. Some traditional Chinese medicine (TCM) compounds can improve obesity by increasing energy consumption. This study aimed to identify and verify TCM compounds that promote adipose thermogenesis to alleviate obesity via transcriptomic analysis, molecular docking, network pharmacology, and Connectivity Map (CMap) analysis. Thirty‐six TCMs related to adipose tissue thermogenesis were first collected to generate transcriptomic data. Then a ranking method based on integrated transcriptomic data was used to select emodin from the TCM Da Huang for network pharmacology to explore its potential mechanisms and further experimental validation. CMap analysis of thermogenesis signature genes from ProFAT and GEO datasets identified triptolide as another compound for improving obesity through enhanced thermogenesis. In vitro experiments with 3T3‐L1 cells and in vivo experiments with zebrafish were conducted. The results showed that both emodin and triptolide could upregulate the NAD^+^/NADH ratio, increase AMPK and SIRT1 expression, suppress lipid accumulation, and promote lipolysis, as verified by both in vitro and in vivo experiments. They also upregulated thermogenesis‐related genes (e.g., UCP1, PGC1α, and PRDM16), lipolysis‐related genes (e.g., PKA, ATGL, and HSL), mitochondrial biogenesis‐related genes (e.g., NRF1, NRF2, and TFAM), and β‐oxidation‐related genes (e.g., CPT1α and CPT1β). Meanwhile, they downregulated lipogenesis‐related genes such as FASN, SREBP1, and ACC. These results provide a basis for understanding the effects of emodin and triptolide on obesity. The findings highlight the effectiveness of combined screening approaches in exploring compounds with beneficial metabolic effects.

**Abbreviations**
AAALAC: association for assessment and accreditation of laboratory animal care
ACC: acetyl‐CoA carboxylase
ADME: absorption, distribution, metabolism, and excretion
AMPK: AMP‐activated protein kinase
ATGL: adipose triglyceride lipase
AVE: average
BAT: brown adipose tissue
C/EBPα: CCAAT/enhancer‐binding protein alpha
C/EBPβ: CCAAT/enhancer‐binding protein beta
CCK‐8: cell counting kit‐8
CIDEA: cell death‐inducing DFF45‐like effector A
CMap: connectivity map
CNKI: China national knowledge infrastructure
CPT1α: carnitine palmitoyltransferase 1 alpha
CPT1β: carnitine palmitoyltransferase 1 beta
DEGs: differentially expressed genes
FASN: fatty acid synthase
GEO: gene expression omnibus
GI: gastrointestinal
GO: gene ontology
GSEA: gene set enrichment analysis
HSL: hormone‐sensitive lipase
IACUC: institutional animal care and use committee
IC_50_
: half‐maximal inhibitory concentration
iDEP: integrated differential expression and pathway analysis
KEGG: kyoto encyclopedia of genes and genomes
LDL‐C: low‐density lipoprotein cholesterol
MTC: maximum testing concentration
NAMPT: nicotinamide phosphoribosyltransferase
NRF1: nuclear respiratory factor 1
NRF2: nuclear respiratory factor 2
PAR2: protease‐activated receptor 2
PGC‐1α: peroxisome proliferator‐activated receptor gamma coactivator 1‐alpha
PKA: protein kinase A
PPARα: peroxisome proliferator‐activated receptor alpha
PPARγ: peroxisome proliferator‐activated receptor gamma
ProFAT: profiling of fat tissue types
PRDM16: PR domain containing 16
SIRT1: sirtuin 1
SREBP1: sterol regulatory element‐binding protein 1
TC: total cholesterol
TCM: traditional Chinese medicine
TFAM: mitochondrial transcription factor A
UCP1: uncoupling protein 1
WAT: white adipose tissue

## 1 Introduction

Obesity has become a public health issue in China. The latest national survey showed that over half of Chinese adults are overweight or obese (Pan et al. 2021). Obesity is a significant risk factor for type 2 diabetes mellitus (T2DM) (Kusminski et al. 2016). Obesity and T2DM represent critical public health challenges due to their link with life‐threatening diseases, including cardiovascular diseases and cancers (Ruze et al. 2023). Obesity and the transition to T2DM may be partly attributed to alterations in adipose tissue composition and function (Cheng, Wang, et al. 2021). Brown adipose tissue (BAT) dissipates chemical energy by uncoupling mitochondrial respiration to generate heat. Adipose thermogenesis can significantly increase energy expenditure in the human body, making it a promising therapeutic target for treating obesity and T2DM (Betz and Enerback 2018; Cheng et al. 2024). Before the introduction of Western medicine, Chinese people had relied on traditional Chinese medicine (TCM) for thousands of years to treat diseases and promote health. Recently, a large amount of data has demonstrated the long‐term effectiveness and low adverse effects of TCM on various illnesses, including obesity and T2DM. Several natural compounds, including artemisinin, capsaicin, celastrol, curcumin, berberine, and ginsenosides, have been widely evaluated for their properties and potential to combat obesity and other metabolic diseases (Li et al. 2019; Quan et al. 2020).

Drug perturbation expression profiling has garnered increasing attention as an effective drug development technique. Connectivity Map (CMap) is a database of gene expression profiles from cultured human cell lines treated with FDA‐approved drugs. A pattern‐matching algorithm was applied to mine these data, allowing for the comparison of gene expression signatures to investigate relationships among drugs, genes, and diseases (Lv et al. 2017). The CMap database has been successfully applied to research several TCM compounds. For instance, Liu et al. employed CMap to identify celastrol, a natural compound from thunder god vine, which can increase leptin sensitivity to suppress food intake and reduce the body weight of obese mice (Liu et al. 2015). Similarly, Chen et al. used transcriptomic data of white adipose tissue (WAT) and BAT of cold‐exposed mice to establish the gene signature of cold‐induced gene expression profiles using CMap (Chen et al. 2021). With the help of the LiP‐MS (Limited Proteolysis‐Mass Spectrometry) experiment, they demonstrated that hyperforin triggers thermogenesis in adipose tissue to inhibit obesity via the Dalt‐AMPK signaling pathway. Combining the CMap database and other bioinformatics analyses to elucidate gene expression profiles, drug targets/pathways, and mechanisms of action of Chinese herbs and TCM compounds will contribute to the pharmacological prediction and effectiveness of drug discovery.

Network pharmacology, integrating systems biology and bioinformatics, explores TCM mechanisms in diseases. It focuses on the interaction of multicomponent, multitarget, and multifunctional pathways at various biological levels (Hopkins 2008; Zhai et al. 2025). While it is helpful for preliminary screening, wet lab experiments are necessary for validation. Molecular docking predicts compound‐protein interactions, aiding in drug design (Agu et al. 2023). TCM's unique system, characterized by multiple components and targets, presents modernization challenges. Recent studies use network pharmacology and molecular docking to analyze TCM pharmacological activities (Dai et al. 2020). Dai et al. identified five key targets for Fu Ling Gui Zhi Decoction in treating breast cancer, analyzed the binding modes of these targets, and elucidated the “monarch, minister, assistant, and envoy” relationships among the TCM components.

The AMPK (AMP‐activated protein kinase)/SIRT1 (Sirtuin 1) pathway plays a crucial role in energy metabolism, particularly in processes such as thermogenesis, lipolysis, mitochondrial biogenesis, and β‐oxidation (Ruderman et al. 2010). AMPK serves as a key sensor of cellular energy status and is activated under low energy conditions, such as fasting or exercise. SIRT1, a NAD^+^‐dependent deacetylase, regulates metabolic homeostasis (Majeed et al. 2021). Both AMPK and SIRT1 are interconnected in their regulatory functions. AMPK activation can increase NAD^+^ levels by upregulating NAMPT (nicotinamide phosphoribosyltransferase), a key enzyme in the NAD^+^ salvage pathway, enhancing SIRT1 activity. SIRT1 can enhance the activity of AMPK by deacetylating PGC‐1α (Peroxisome Proliferator‐Activated Receptor Gamma Coactivator 1‐Alpha). PGC‐1α is an essential coactivator involved in regulating gene expression related to energy metabolism. Higher NAD^+^ levels activate SIRT1, promoting metabolic benefits such as improved insulin sensitivity and reduced inflammation, which are beneficial in conditions like diabetes and obesity (Ruderman et al. 2010).

In this study, we first conducted a literature search of TCM or their compounds that showed potential to enhance adipose tissue thermogenesis (Figure 1). Meanwhile, thermogenesis marker genes were collected from databases. After treating cells with selected TCMs and performing RNA sequencing, we input gene expression profiles into the ProFAT to predict the thermogenesis probability of TCMs for further experiments (Cheng et al. 2018). Simultaneously, CMap analysis was performed to identify the TCM compounds. Finally, the screened compounds were included for experimental validation. This study aimed to identify potential compounds that can help promote adipose tissue thermogenesis to alleviate obesity.

**FIGURE 1:**
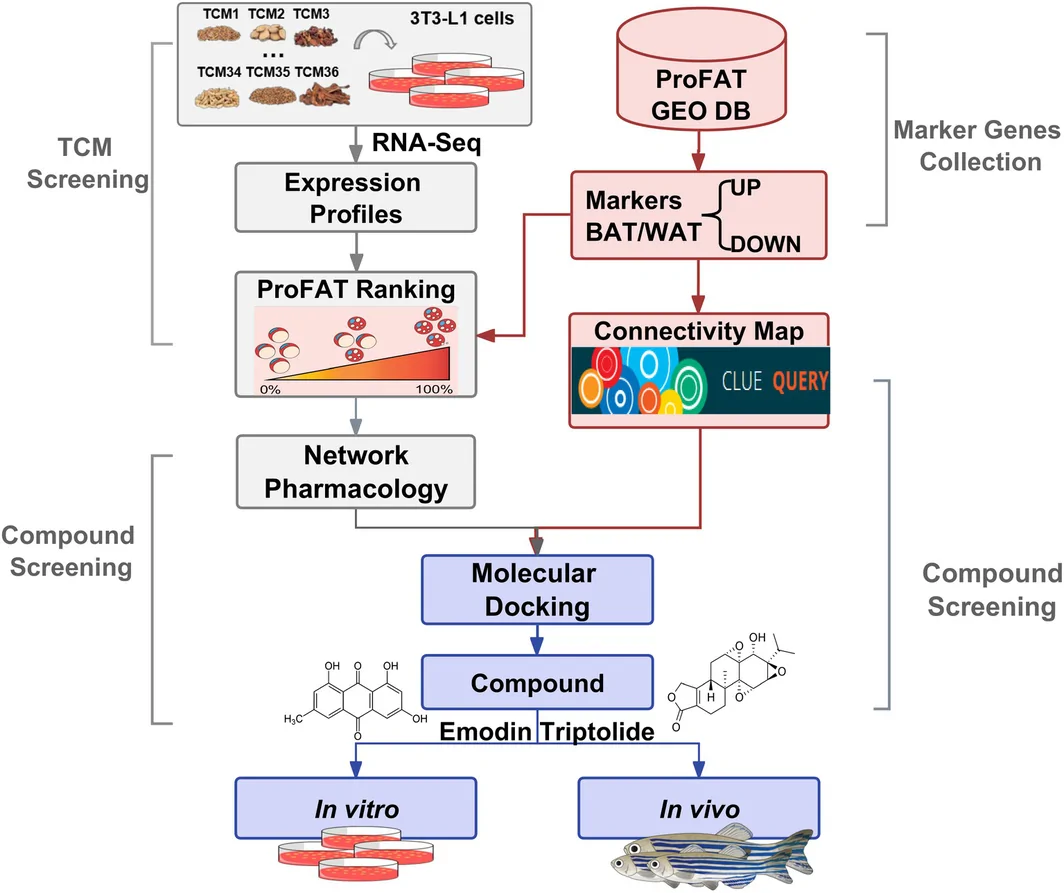
Workflow for the screening of compounds promoting adipose thermogenesis.

## 2 Materials and Methods

### 2.1 TCM Collection

To identify English and Chinese peer‐reviewed literature reporting potential TCMs or their compounds for enhancing thermogenesis, we searched in PubMed, Google Scholar, and the Chinese National Knowledge Infrastructure (CNKI). We utilized the keywords “(thermogenesis [OR] obesity) [AND] (TCM [OR] natural product)” in PubMed and Google Scholar to collect TCMs related to adipose thermogenesis. Finally, we compiled 36 TCMs for the cell experiments (Table S1).

### 2.2 3T3‐L1 Cell Differentiation

3T3‐L1 cells were purchased from Cellcook Biotech Co. Ltd. (Guangzhou City, China) and cultured in Dulbecco's Modified Eagle Medium (DMEM, Cat. No. CM2007) supplemented with 10% newborn calf serum (NBCS) at 37°C with 5% CO_2_. Once the seeded cells reached 100% confluence, the preadipocyte medium was replaced, and the cells were allowed to grow for an additional 48 h. Following this, the cells were incubated with the differentiation medium composed of 90% DMEM, 10% fetal bovine serum (FBS), 1.0 μM dexamethasone (DEX, Cat. No. D137736, Aladdin), 5.0 mM 3‐isobutyl‐1‐methylxanthine (IBMX, Cat. No. I106812, Aladdin), and 10 μg/mL insulin for 48 h. After 48 h of differentiation, the differentiation medium was replaced with the maintenance medium containing 90% DMEM, 10% FBS, and 10 μg/mL insulin. The maintenance medium was refreshed every 48 h. The cells were confirmed to be fully differentiated by the 10th day (Figure S1a).

### 2.3 CCK‐8 Assay

Cells were seeded in a 96‐well plate at a density of 5000 cells per well. The TCM concentrated granules (E‐Fang Pharmaceutical Co. Ltd., Guangdong Province, China) and compounds (emodin: Cat. No. E106693, Lot. No. C2225043, Aladdin; triptolide: Cat. No. T107399, Lot. No. L2128200, Aladdin) were diluted in the culture medium. After a 24‐h incubation, the CCK‐8 working solution was added to each well. The plates were then incubated for 2 h before measuring the optical density (OD) values at 450 nm using a Bio‐Tek Microplate Reader.

### 2.4 Library Preparation, mRNA‐Sequencing, and Data Analysis

Library preparation and sequencing methods were slightly modified based on the previously reported method (Li et al. 2023). Total RNA was isolated with TRIzol (Cat. No. 15596026, Invitrogen) and assessed for integrity using the 2100 Agilent Bioanalyzer (Agilent, USA). The library preparation started with 1 μg of total RNA for each sample. Rolling circle amplification (RCA) was subsequently conducted to generate DNA nanoballs (DNBs). Paired‐end reads of 100 bp (PE100) were sequenced on the MGISEQ‐2000RS platform (MGI, Shenzhen, China).

For differential expression analysis, we utilized iDEP (http://ge‐lab.org/idep/), an integrated tool for differential expression and pathway analysis (Ge et al. 2018). Differentially expressed genes (DEGs) were selected based on a false discovery rate (FDR) of ≤ 0.05 and an absolute fold change of ≥ 2. For enrichment analysis, we used several tools, including Metascape (Zhou et al. 2019), GSEA (Subramanian et al. 2005), and ClueGO (Bindea et al. 2009), to validate and interpret the enrichment results.

### 2.5 Thermogenesis Marker Gene Collection

BAT and WAT marker genes were collected from the Gene Expression Omnibus (GEO) and ProFAT (Cheng et al. 2018) (http://ido.helmholtz‐muenchen.de/ProFAT/). The GEO database was queried, and five datasets from *Mus musculus* , namely GSE131861 (Wang et al. 2019), GSE86338 (Bai et al. 2017), GSE98132 (Hepler et al. 2017), GSE73266 (Shinozaki et al. 2016), and GSE37248 (van Zutphen et al. 2019), were finally selected (Table S2).

### 2.6 TCM Thermogenesis Probability Ranking Strategy

To rank the TCM thermogenesis probability, we firstly generated expression profiles from cells treated with various TCMs. The transcriptomic data from 36 TCMs were input into the ProFAT, a tool designed to predict the thermogenesis probability of microarray data and RNA‐seq data (Cheng et al. 2018). These TCMs were ranked based on the ProFAT score calculated as ProFAT score = Browning_AVE (H + L + M)/Browning_AVE (CTRL1 + CTRL2) (Figure 3A). Subsequently, transcriptomic data for each TCM were input into iDEP to obtain the DEGs for each TCM compared with the control. DEGs from the analysis of GEO datasets were then used to intersect with the DEGs from TCM RNA‐seq (upregulated GEO_DEGs ∩ upregulated TCM_DEGs; downregulated GEO_DEGs ∩ downregulated TCM_DEGs). The numbers of intersected DEGs (Up + Down) were used to rank the TCM thermogenesis probability. Finally, the ProFAT score ranking and DEGs ranking of TCMs were integrated to screen the TCM candidates.

### 2.7 Network Pharmacology Analysis of TCM Da Huang

Network pharmacology analysis was performed to identify hub compounds in TCM Da Huang. The TCM compounds were collected from multiple databases: TCMSP (Ru et al. 2014) (https://old.tcmsp‐e.com/tcmsp.php), ETCM (Xu et al. 2018) (http://www.tcmip.cn/ETCM/), BATMAN (Liu et al. 2016) (http://bionet.ncpsb.org.cn/batman‐tcm/), and TCM Database@Taiwan (Chen 2011) (http://tcm.cmu.edu.tw/). We obtained each compound's PubChem ID and SMILES information and imported the data into Gephi to calculate the structure similarity (Bastian et al. 2009). Compounds were then divided into different modules based on their chemical structures. Putative protein targets were collected from the BioAssay results from PubChem (https://pubchem.ncbi.nlm.nih.gov), focusing solely on active interactions between compounds and human proteins (Kim et al. 2023). Targets were also collected from the SEA (Keiser et al. 2007) (https://sea.bkslab.org/).

### 2.8 ADME Analysis of the Compounds From TCM Da Huang

The characteristic compounds of TCM Da Huang were evaluated for drug activity through ADME analysis. Canonical SMILES of compounds were input into SwissADME (Daina et al. 2017) (http://www.swissadme.ch/) and ADMETlab 2.0 (Xiong et al. 2021) (https://admetmesh.scbdd.com/) to obtain information on blood–brain barrier (BBB) permeability, Lipinski rule of five (molecular weight < 500, number of hydrogen bond acceptors < 10, number of hydrogen bond donors < 5, lipophilicity‐iLogP < 5, and number of rotatable bond < 10), gastrointestinal (GI) absorption, and bioavailability scores. Only compounds that adhered to Lipinski's, Ghose's, Veber's, Egan's, and Muegge's rules and exhibited high GI absorption, BBB permeability (> 0.3), and bioavailability score (> 0.3) were included.

### 2.9 Compound Target Interaction Prediction by Molecular Docking

The 3D structures of emodin and triptolide in SDF format were downloaded from PubChem and imported into ChemBio3D Ultra 14.0 for energy minimization. The optimized molecules were then imported into AutodockTools (v1.5.6) for hydrogen addition, charge calculation, charge distribution, and rotatable bond setting, and they were saved in “pdbqt” format. The PPARγ (PDB ID: 7AWC) (Willems et al. 2021) and PAR2 (PDB ID:5NDZ) (Cheng et al. 2017) protein structures were downloaded from the Protein Data Bank (Rose et al. 2017) (PDB, http://www.rcsb.org/pdb). Using PyMOL, protein crystallization water and original ligands were removed from the protein structure. The protein structures were imported into AutodockTools (v1.5.6) for hydrogen addition, charge calculation, charge distribution, and atomic type, and they were saved in “pdbqt” format. We used POCASA 1.1 (Yu et al. 2010) to predict the protein binding site and AutoDock Vina 1.1.2 for docking. Finally, the interaction of the docking results was analyzed using PyMOL 2.3.0.

### 2.10 Quantitative Real‐Time PCR Analysis

qPCR was conducted following the procedures reported in our previous research (Li et al. 2024). Total RNA was extracted using TRIzol (Cat. No. 15596026, Invitrogen) according to the manufacturer's instructions. First‐strand cDNA synthesis was performed using 1 μg of total RNA with SuperScript III Reverse Transcriptase (Cat. No. 18080‐044, Invitrogen) in a 20 μL reaction volume. The reverse transcription was performed at 50°C for 60 min, followed by inactivation at 70°C for 15 min. Each reaction was carried out in triplicate in a 20 μL final volume, containing 10 μL of SYBR Green PCR Master Mix, 0.5 μM of each primer, and 2 μL of cDNA template. Finally, the assay was performed on an Applied Biosystems Quant Studio 6.0 (Thermo Fisher Scientific, USA). GAPDH served as the reference gene. The primer sequences used in this study can be found in Table S3. Gene expressions were analyzed using the 2^−ΔΔCT^ method.

### 2.11 Western Blot

Western blot was conducted following the procedures reported in our previous research (Li et al. 2024). Cells were lysed using RIPA buffer (Cat. No. P0013C, Beyotime, China), and protein concentration was measured using the BCA protein assay (Cat. No. P0012S, Beyotime, China). Then, 20 μg of total protein was separated by 10% SDS‐PAGE and transferred onto polyvinylidene difluoride membranes (PVDF; Millipore, USA) using a wet transfer system at 300 mA for 1.5 h at 4°C. After transfer, the membranes were blocked with 5% BSA for one hour at room temperature to reduce non‐specific binding. After blocking, membranes were incubated overnight at 4°C with the primary antibodies listed in Table S4. The membranes were then incubated with the horseradish peroxidase (HRP)‐conjugated secondary antibodies (Cat. No. A0216, 1:2000, Beyotime, China) for one hour at room temperature. Finally, the protein bands were visualized using enhanced chemiluminescence (ECL) reagent (Cat. No. P0018, Beyotime, China) and detected using a chemiluminescence imaging system. The intensity of the protein bands was quantified using ImageJ software, and the results were normalized to GAPDH.

### 2.12 Oil Red O Staining in 3T3‐L1 Cells

Upon completion of differentiation, the culture medium was removed, and cells were fixed with 4% formaldehyde (Cat. No. P0099, Beyotime, China) for 10 min. Following fixation, the cells were washed, and the Oil Red O working solution (Cat. No. C0158S, Beyotime, China) was evenly applied to cover them. After incubation for 20 min, the cells were washed with washing buffer for 30 s and subsequently rinsed with PBS for 20 s. The cells were then covered with PBS and observed under a microscope.

### 2.13 NAD ^+^/NADH in 3T3‐L1 Cells

According to the instructions of the NAD^+^/NADH Assay Kit (Cat. No. S0175, Beyotime, China), 200 μL of NAD^+^/NADH extraction solution was firstly added to lyse 3T3‐L1 cells. The lysates were centrifuged at 12,000 × g for 10 min. The sample was heated in a 60°C water bath for 30 min, followed by the addition of 90 μL of alcohol dehydrogenase (ADH) working solution. Then, the mixture was incubated for 10 min at 37°C, protected from light. Subsequently, 100 μL of color reagent was added, and the mixture was thoroughly mixed. The sample was then incubated for another 10 min at 37°C without exposure to light before measuring the OD at 450 nm.

### 2.14 Animal Preparation

Zebrafish were raised in fish breeding water at 28°C. The zebrafish experiments were accredited by the Association for Assessment and Accreditation of Laboratory Animal Care (AAALAC) International (SYXK2022‐0004). This study was approved by the Institutional Animal Care and Use Committee (IACUC) at Hunter Biotechnology Inc. (No.: 001458). Before conducting the assays, we determined the maximum testing concentrations for the lipolysis, fat absorption, and NAD^+^/NADH assays in the zebrafish model (Tables S5–S7).

### 2.15 Lipolysis Assay in Zebrafish

To conduct the lipolysis assay in zebrafish, two‐day‐post‐fertilization (dpf) albino zebrafish were randomly selected and placed into six‐well plates with 30 zebrafish per well. Emodin and triptolide were administered at various concentrations: emodin at 0.098, 0.195, and 0.391 μg/mL, and triptolide at 0.024, 0.049, and 0.098 μg/mL. A positive control group was treated with 11.4 μg/mL resveratrol, and a normal control group was included. After treatment at 28°C for one day, the zebrafish in each group were treated with water‐solubilized Nile Red dye. Treatment continued at 28°C until the zebrafish reached 4 dpf. Then, ten randomly selected zebrafish from each group were photographed under a fluorescence microscope. The fluorescence intensity of the zebrafish yolk sac fat was analyzed using NIS‐Elements D 3.20 advanced image processing software (Figure S1b).

### 2.16 Fat Absorption Assay in Zebrafish

For the fat absorption assay, 5 dpf albino zebrafish were randomly selected and placed in beakers with 30 zebrafish per beaker. Emodin and triptolide were water‐dissolved and administered at various concentrations: emodin at 0.098, 0.195, and 0.391 μg/mL, and triptolide at 0.049, 0.098, and 0.195 μg/mL. A positive control group treated with 15.0 μg/mL orlistat, a normal control group, and a model control group were also established. After treatment at 28°C for one hour, except for the normal control group, all treatment groups were fed with egg yolk powder to develop a food fat absorption model in zebrafish. Treatment continued at 28°C for one additional day. Subsequently, Oil Red O was used for whole‐body fat staining. After staining, ten zebrafish from each group were randomly selected and photographed under a dissecting microscope. The staining intensity in the intestine and tail vasculature was analyzed using the NIS‐Elements D 3.20 advanced image processing software (Figure S1c).

### 2.17 NAD ^+^/NADH Assay in Zebrafish

Six hpf (hours post‐fertilization) wild‐type AB zebrafish were randomly selected and placed in six‐well plates, with 30 zebrafish in each well for each group. Emodin and triptolide were dissolved in water and administered at different concentrations: emodin at 0.024, 0.049, and 0.098 μg/mL, and triptolide at 0.012, 0.024, and 0.049 μg/mL. Additionally, a normal control group was established. After treatment at 28°C for 6 days, zebrafish samples were collected according to the instructions of the NAD(H) content assay kit (Solarbio, China), and the NAD^+^, NADH contents, and NAD^+^/NADH ratio of zebrafish were detected using an ELISA reader (Figure S1d).

### 2.18 Statistical Analysis

All experimental data were presented as the mean ± SD from three independent experiments and analyzed using GraphPad Prism 9 software. A statistical analysis of the data was performed using the Student's *t*‐test. Statistical significance was set at *p* < 0.05.

## 3 Results

### 3.1 TCM Collection and Dosage Determination for In Vitro Experiments

Through the literature survey, we finally compiled a list of 36 TCMs that either nourish qi and strengthen the spleen according to the TCM theory or have been reported to be associated with adipose thermogenesis (Table S1). A high dose of these TCMs was found to significantly suppress the proliferation of 3T3‐L1 cells, exhibiting a dose‐dependent effect (Figure S2). To determine appropriate dosages, we used GraphPad software to calculate the IC_50_ values for each TCM on 3T3‐L1 cells. These IC_50_ values were then used to establish low, medium, and high‐concentration groups for subsequent in vitro experiments (Table S1).

### 3.2 Adipose Thermogenesis Marker Gene Collection From the GEO and ProFAT

Through DEG analysis via iDEP, we identified 227 common DEGs (BAT vs. WAT) across the 28 WAT and 28 BAT mouse tissues from five GEO datasets (Figure 2a, Table S2). Additionally, we found 39 overlapping genes between these DEGs and thermogenesis marker genes from ProFAT (39/59; Figure 2b). The GO enrichment analysis conducted using Metascape revealed that these genes were enriched in processes such as aerobic respiration, tricarboxylic acid cycle, mitochondrial electron transport, and regulation of fatty acid oxidation (Figure 2c). Reactome pathway analysis demonstrated that these genes were enriched in oxidative phosphorylation, fatty acid metabolism, and carbon metabolism (Figure 2d). Furthermore, we performed GO and KEGG enrichment analyses using the Cytoscape plugin ClueGO (Figure 2e,f). The GO enrichment analysis highlighted that these genes were enriched in biological processes such as fatty acid β‐oxidation, electron transport chain, regulation of cold‐induced thermogenesis, and mitochondrial respiratory chain complex assembly. The KEGG enrichment analysis showed that these genes were enriched in pathways such as oxidative phosphorylation, fatty acid degradation, TCA cycle, and PPAR signaling pathway. In Figure 2g, the heatmap was generated using the fold change values from each dataset to visualize the gene expression patterns. These genes exhibit similar expression patterns across different datasets. These findings collectively indicate that these genes are reliable markers of thermogenesis.

**FIGURE 2:**
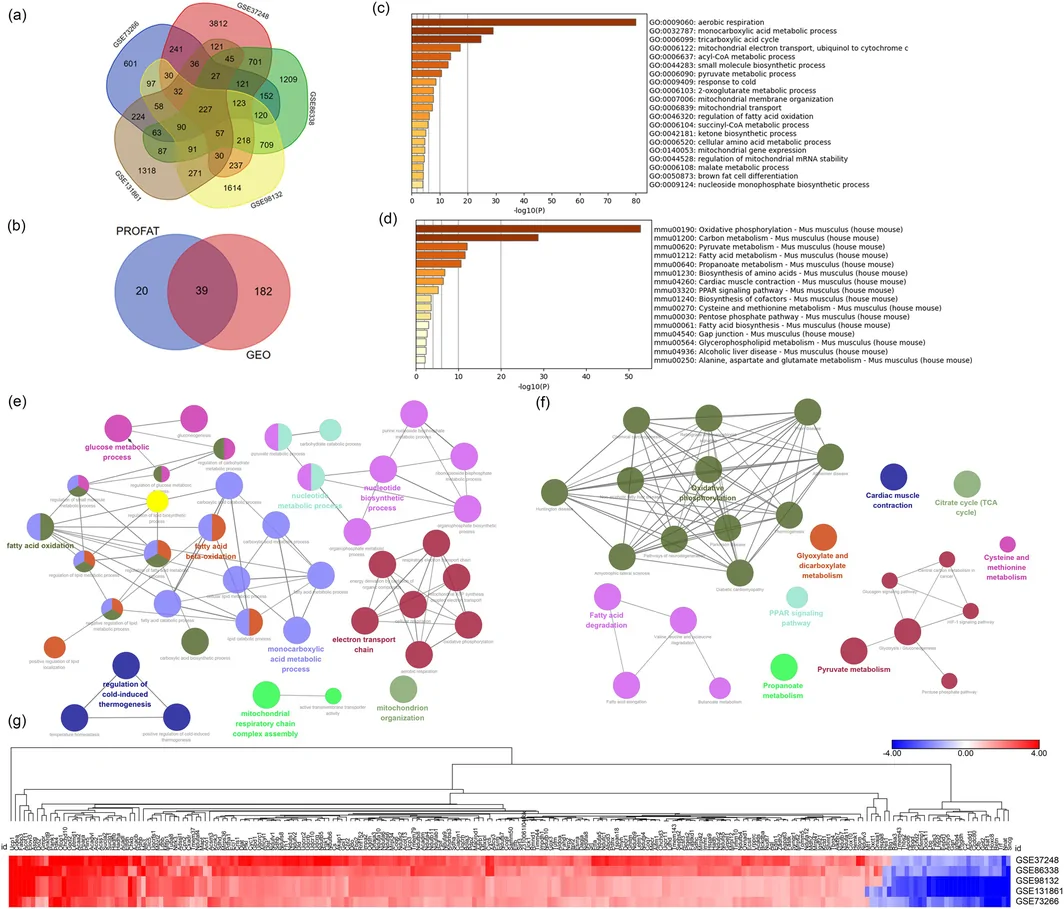
GEO transcriptomic data analysis. (a) Analysis of differentially expressed genes (DEGs) between brown adipose tissue (BAT) and white adipose tissue (WAT) in five GEO datasets. (b) Overlapping genes between DEGs and ProFAT signature genes. GO enrichment (c) and pathway enrichment (Reactome) (d) analysis of the DEGs through Metascape. Network analysis of GO enrichment (e) and KEGG enrichment (f) using ClueGO. (g) Heatmap showing the expression levels of DEGs generated by Morpheus.

### 3.3 TCM Thermogenesis Probability Ranking Based on Transcriptomic Data

The transcriptomic data of 36 TCMs were input into the ProFAT, a tool designed to predict the thermogenesis probability of microarray and RNA‐seq data. Initially, we ranked these TCM agents by their ProFAT scores (Figure 3b). Subsequently, we input TCM transcriptomic data into iDEP to obtain the DEGs for each TCM (TCM vs. CTRL, Table S8). We then intersected the DEGs from the TCM RNA‐seq data with those from the GEO dataset analysis (upregulated GEO_DEGs ∩ upregulated TCM_DEGs; downregulated GEO_DEGs ∩ downregulated TCM_DEGs). The total number of intersected DEGs (Up + Down) was used to rank the TCM thermogenesis probability (Figure 3a,c). Finally, we integrated the ProFAT score ranking and the DEG ranking of the TCMs to obtain the candidate TCM. Through this integrated ranking method, we identified TCM Da Huang as the top TCM, exhibiting the highest probability of promoting thermogenesis (Figure 3d,e).

**FIGURE 3:**
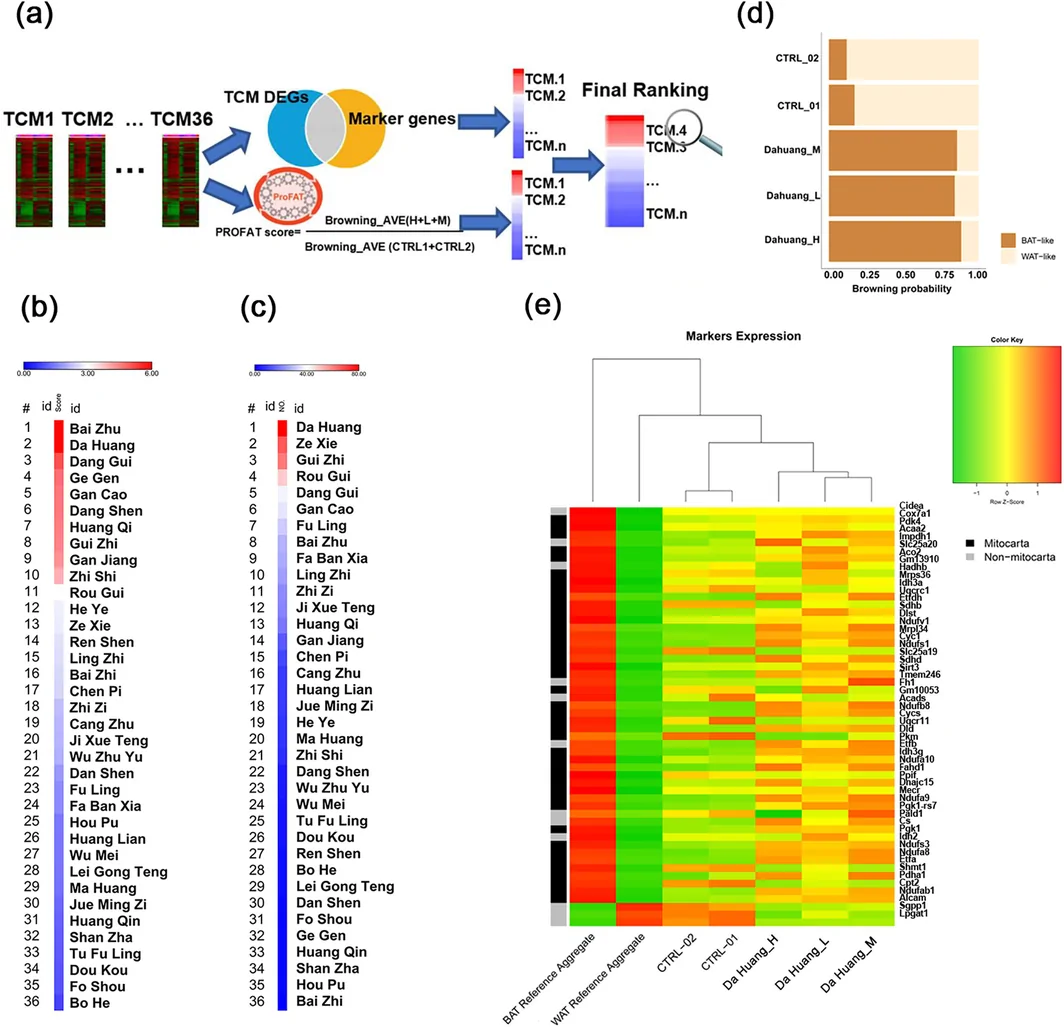
Integrated ranking analysis for thermogenesis probability of 36 TCMs. (a) Overall TCM ranking strategy. (b) ProFAT score‐based ranking strategy. (c) GEO dataset‐based DEGs ranking strategy. (d) Browning score of TCM Da Huang generated by ProFAT. (e) Expression heatmap of thermogenesis marker genes induced by TCM Da Huang generated by ProFAT.

### 3.4 GSEA of TCM Da Huang

To further validate the screening results, we performed GSEA for TCM Da Huang without DEG limitation. The screening parameters included normalized enrichment score > 1, *p*‐value < 0.05, and FDR < 0.25. Our analysis revealed that the upregulated biological processes by Da Huang included glutathione metabolic process, glycolytic process through fructose‐6‐phosphate, positive regulation of triglyceride metabolic process, response to mitochondrial depolarization, mitochondrial translation, mitochondrial respiratory chain complex assembly, and oxidative phosphorylation. In contrast, the downregulated biological processes included transposition and sister chromatid cohesion (Figure 4a). Furthermore, the upregulated Reactome pathways included the metabolism of porphyrins, glutathione conjugation, mitochondrial translation, and insulin receptor recycling. On the other hand, the downregulated pathways were associated with homologous DNA pairing and strand exchange (Figure 4b). To visualize the GSEA results, we used AutoAnnotate, a Cytoscape plugin, to create a network representation of GO enrichment (Kucera et al. 2016). Figure 4c showed that the biological processes enriched by TCM Da Huang could be grouped into several clusters associated with lipid metabolism. These clusters include “negative lipid respiration”, “coupled transport ATP”, “NADH regeneration”, “regulation of lipid triglyceride”, and “nucleoside process monophosphate”.

**FIGURE 4:**
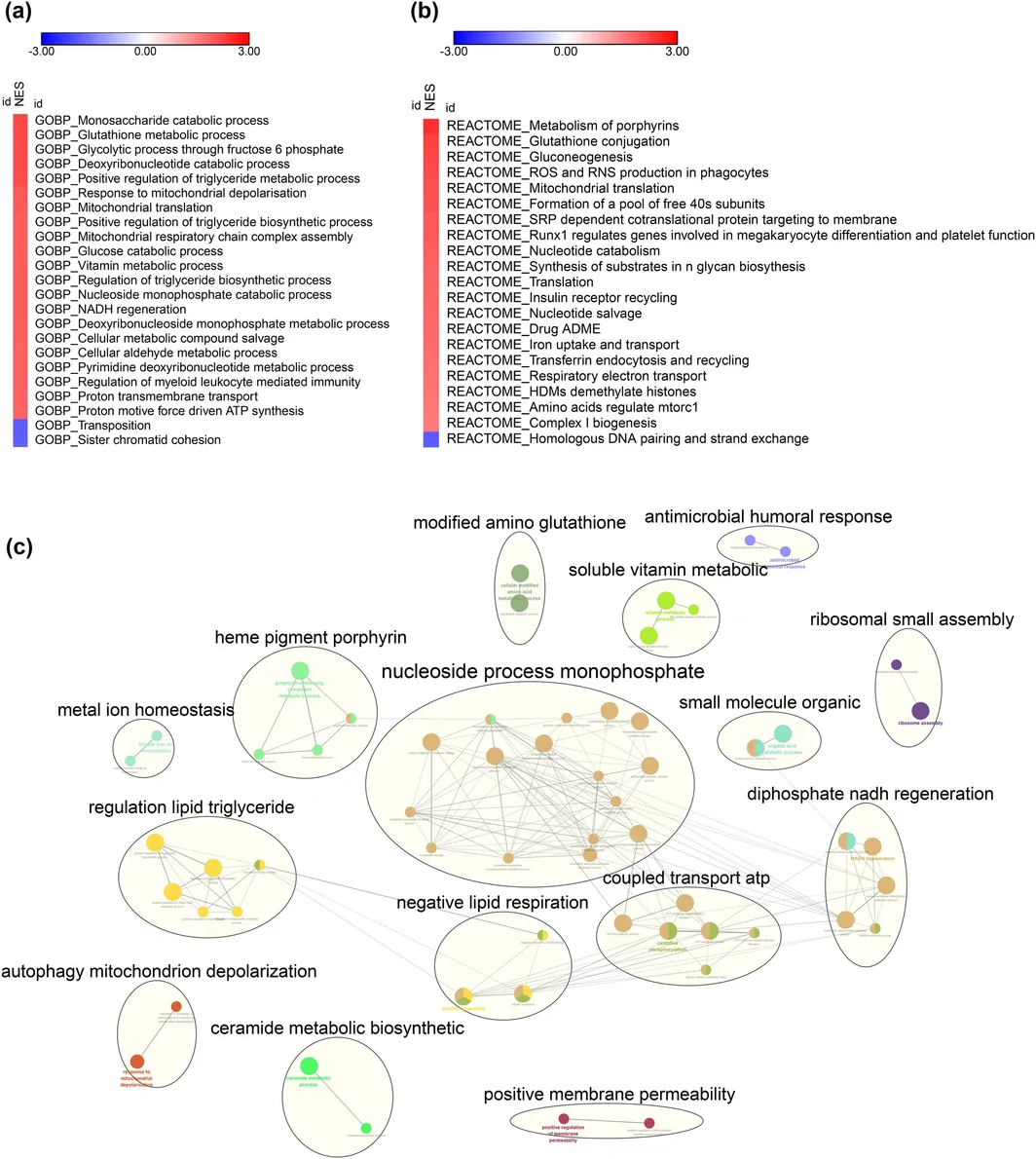
GSEA analysis of transcriptomic data from 3T3‐L1 cells treated with Da Huang. (a) GO enrichment analysis. (b) Reactome enrichment analysis. (c) Network analysis of the GO enrichment results.

### 3.5 Network Pharmacology Analysis of TCM Da Huang

We conducted a network pharmacology analysis of TCM Da Huang by collecting 137 compounds from databases such as TCMSP, ETCM, BATMAN, and TCM Database@TAIWAN (Table S9). These compounds were subjected to structure similarity analysis using Gephi (Table S10), revealing that Module 4 consisted of anthraquinone compounds, which were the main active ingredients in Da Huang (Figure 5a). Additionally, ADME analysis was performed on all 137 compounds, identifying 40 compounds (Table S11) that exhibited remarkable drug properties and adhered to the Lipinski Rule of Five. By intersecting these 40 compounds with Module 4, we finally obtained eight compounds: rhein, aloe‐emodin, chrysophanol‐9‐anthrone, emodin, physcion, chrysophanol, danthron, and emodin anthrone (Figure 5b). Next, through SEA target prediction and PubChem target collection of these eight compounds (Figure S3), emodin was found to act on target PPARγ, which is a transcription factor that is involved in energy metabolism, including adipogenesis, lipid metabolism, and insulin sensitization (Ahmadian et al. 2013). To further validate the interaction, we performed molecular docking between emodin and PPARγ. The binding energy of emodin to PPARγ was −8.1 kcal/mol, demonstrating a strong binding effect. The interaction between emodin and PPARγ was mainly through the formation of hydrogen bonds and hydrophobic interactions. Specifically, emodin formed hydrogen bonds with GLU‐259 and SER‐342, with bond lengths of 3.0 and 2.9 Å, respectively. Additionally, hydrophobic interactions were observed with ILE‐341, ARG‐288, and ILE‐281 (Figure 5c). These findings suggest that emodin is a promising compound for further investigation in the context of metabolic regulation.

**FIGURE 5:**
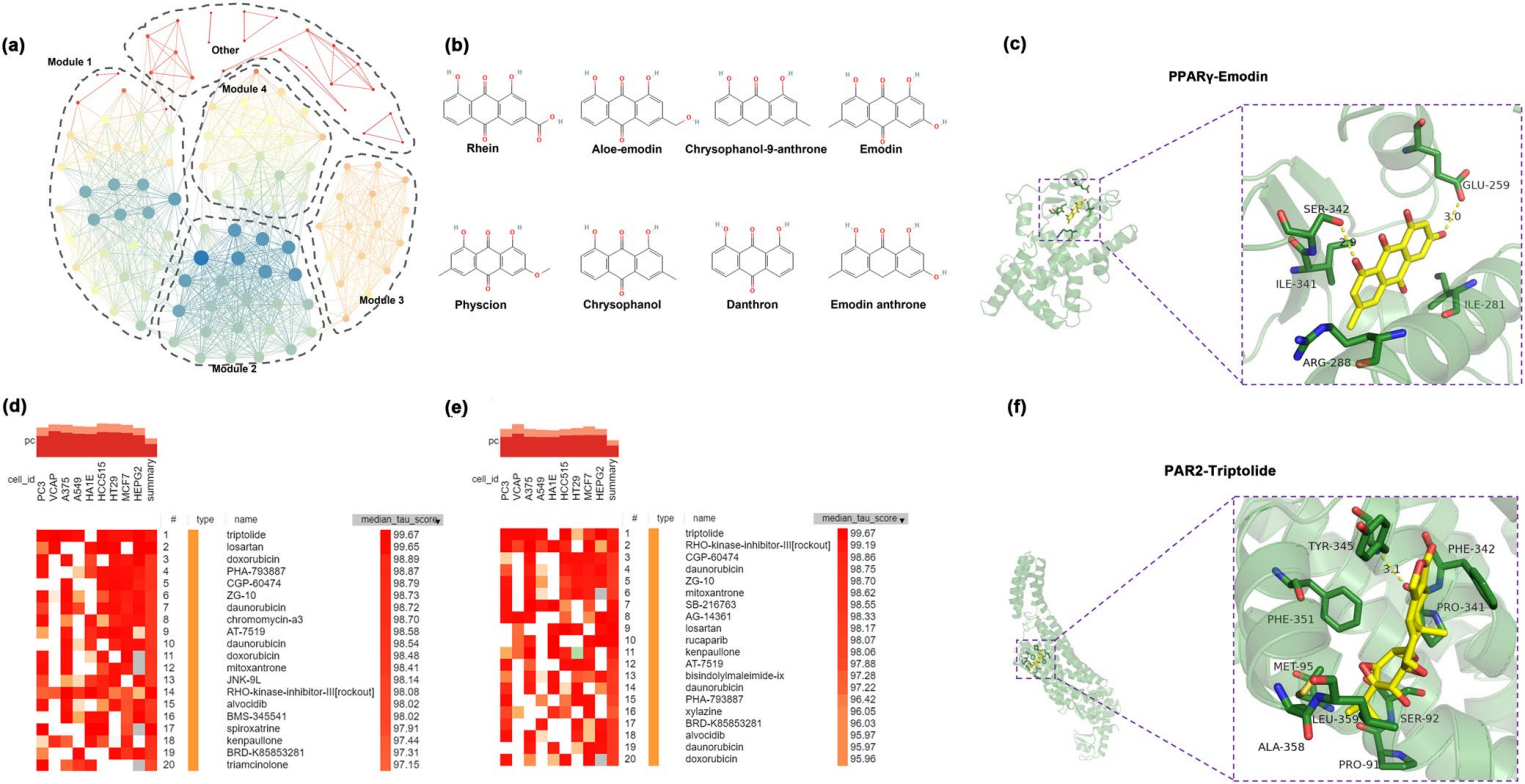
Compound screening by network pharmacology analysis and CMap analysis. (a) Chemical structural similarity analysis of compounds from TCM Da Huang generated by Gephi. (b) Eight compounds were screened from the intersection of 40 compounds that obey the Lipinski Rule of Five and anthraquinone (Module 4 in a). (c) Molecular docking of compound emodin and protein PPARγ. (d and e) CMap analysis using thermogenesis marker genes from ProFAT (d) and GEO (e). (f) Molecular docking of compound triptolide and protein PAR2.

### 3.6 CMap Analysis of the Thermogenesis Marker Genes

To obtain compounds showing similar expression patterns to the browning adipocyte phenotype, thermogenesis‐marker genes from ProFAT and GEO were used as transcriptional profiles to query CMap, respectively. Only compounds with a connectivity score of 90 or higher were selected. Through the CMap analysis of these genes, we obtained two compound lists (Figure 5d,e). Among the identified compounds, triptolide stands out as it is a major active component purified from the extracts of *Tripterygium wilfordii* Hook F. Triptolide is known for its multiple pharmacological activities, including anti‐inflammatory, immune modulation, antiproliferative, and pro‐apoptotic activity (Q. Liu 2011). Using the SEA target prediction, triptolide was predicted to act on target PAR2 (protease‐activated receptor 2), a G protein‐coupled receptor that is involved in promoting diet‐induced obesity, 3T3‐L1 preadipocyte differentiation and high‐fat diet‐induced hepatic steatosis (Badeanlou et al. 2011; Kim et al. 2021; Wang, Liang, et al. 2020). Molecular docking between triptolide and PAR2 revealed a binding energy of −8.2 kcal/mol, indicating a strong binding interaction. The interaction is primarily mediated through the formation of hydrogen bonds and hydrophobic forces. Specifically, triptolide forms a hydrogen bond with TYR‐345, with a hydrogen bond length of 3.1 Å. Additionally, it exhibits hydrophobic interactions with PHE‐342, PRO‐341, SER‐92, PRO‐91, LEU‐359, ALA‐358, MET‐95, and PHE‐351 (Figure 5f). Therefore, triptolide appears to be a promising compound for further investigation in the context of metabolic regulation.

### 3.7 Emodin and Triptolide Inhibit Lipid Accumulation and Enhance the Metabolic Rate in 3T3‐L1 Cells

We first conducted the CCK‐8 assay to determine the effects of emodin and triptolide on the viability of 3T3‐L1 cells (Figure 6b,g). The results demonstrated that emodin concentrations exceeding 25 μM significantly inhibited cell growth, while triptolide concentrations above 20 nM had a similar inhibitory effect. Based on these findings, we selected concentrations of 5, 10, and 25 μM for emodin treatment and 5, 10, and 20 nM for triptolide treatment. Oil Red O staining showed that as the emodin and triptolide concentrations increased, the size and quantity of lipid droplets significantly decreased (Figure 6a,f). These results indicate that emodin and triptolide effectively reduced the lipid content in adipocytes. For the NAD^+^/NADH assay, we observed that treatment with emodin or triptolide significantly increased the NAD^+^ content at 5 and 10 μM emodin, and 5 and 10 nM triptolide (*p* < 0.001; Figure 6c,h). Meanwhile, the NADH content significantly decreased at an emodin concentration of 25 μM (*p* < 0.01), and triptolide concentrations of 10 and 20 nM (*p* < 0.01; Figure 6d,i). This decrease in NADH content, combined with the simultaneous increase in NAD^+^ content, ultimately led to a significant increase in the NAD^+^/NADH ratio, especially at emodin concentrations of 10 and 25 μM (*p* < 0.001), and triptolide concentrations of 10 and 20 nM (*p* < 0.001; Figure 6e,j). The elevated NAD^+^/NADH ratio suggests that emodin and triptolide treatment could substantially enhance the metabolic rate of adipocytes.

**FIGURE 6:**
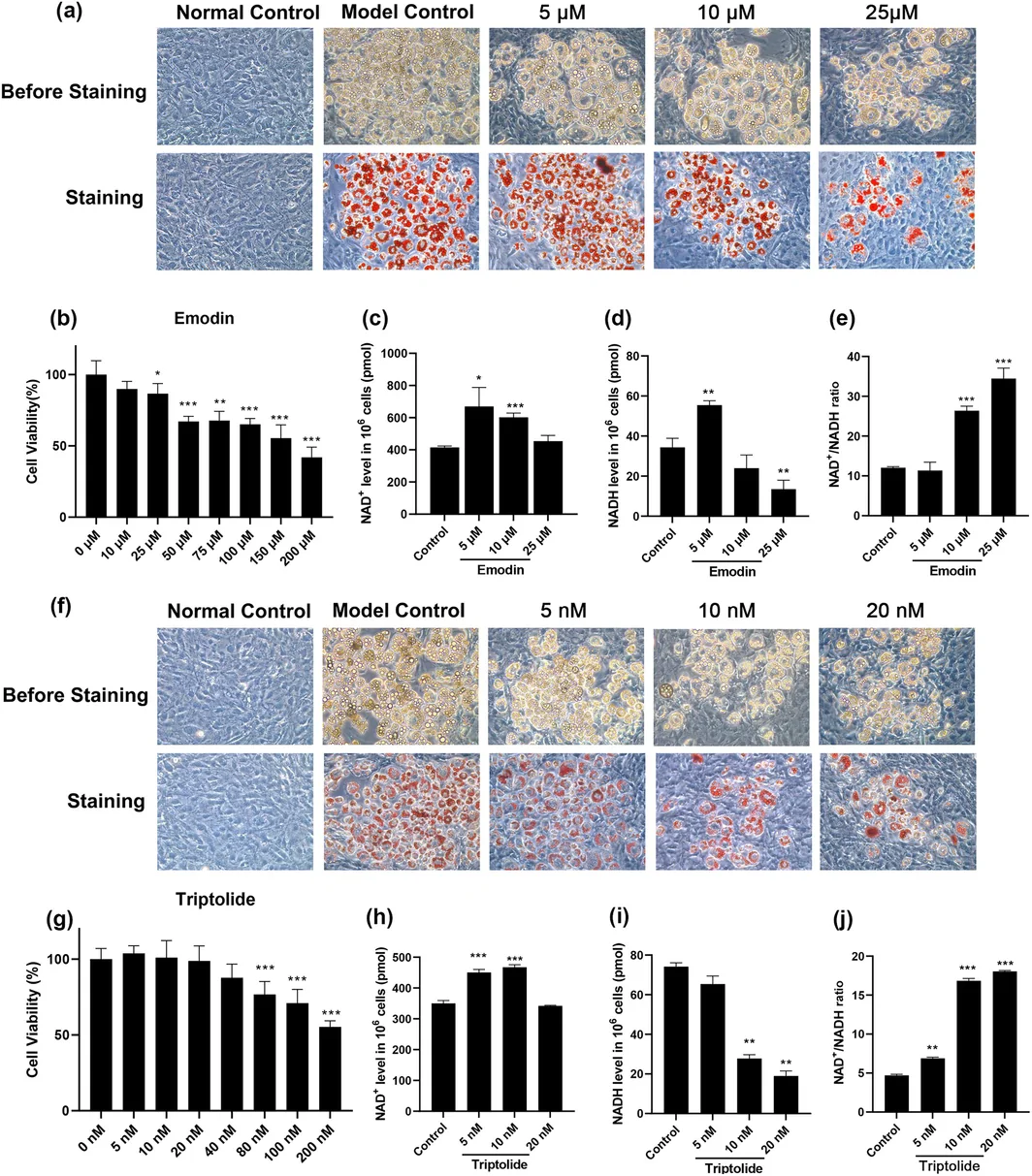
Emodin and triptolide promoted lipolysis and enhanced the metabolic rate in mature 3T3‐L1 adipocytes. (a) Oil red O staining of mature 3T3‐L1 adipocytes treated with emodin at concentrations of 5, 10, and 25 μM. (b) Cell viability of mature 3T3‐L1 adipocytes after emodin treatment at concentrations of 0, 10, 25, 50, 75, 100, 150, and 200 μM. *p < 0.05 vs. the 0 μM treatment, **p < 0.01 vs. the 0 μM treatment, ***p < 0.001 vs. the 0 μM treatment. Detection of the NAD^+^ level (c), NADH level (d), and NAD^+^/NADH ratio (e) in mature 3T3‐L1 adipocytes after emodin treatment at concentrations of 5, 10, and 25 μM. *p < 0.05 vs. the control group, **p < 0.01 vs. the control group, ***p < 0.001 vs. the control group. (f) Oil red O staining of mature 3T3‐L1 adipocytes treated with triptolide at concentrations of 5, 10, and 20 nM. (g) Cell viability of mature 3T3‐L1 adipocytes after triptolide treatment at concentrations of 0, 5, 10, 20, 40, 80, 100, and 200 nM. ***p < 0.001 vs. the 0 nM treatment. Detection of the NAD^+^ level (h), NADH level (i), and NAD^+^/NADH ratio (j) in mature 3T3‐L1 adipocytes after triptolide treatment at concentrations of 5, 10, and 20 nM. *p < 0.05 vs. the control group, **p < 0.01 vs. the control group, ***p < 0.001 vs. the control group.

### 3.8 Emodin and Triptolide Promote Lipolysis, Inhibit Fat Absorption, and Upregulate NAD ^+^/NADH Ratio in Zebrafish

In the lipolysis assay conducted in the zebrafish model, when compared with the normal control group, 0.195 and 0.391 μg/mL of emodin significantly decreased the fluorescence intensity of yolk‐sac adipose tissue in zebrafish (*p* < 0.001; Figure 7a,c). For triptolide treatment, concentrations of 0.024, 0.049, and 0.098 μg/mL also significantly decreased the fluorescence intensity (*p* < 0.05 at 0.024 and 0.049 μg/mL, *p* < 0.001 at 0.098 μg/mL; Figure 7h,j). These results indicate that both emodin and triptolide could dramatically promote lipolysis. In the fat absorption experiment, compared with the model control group, emodin at concentrations of 0.195 and 0.391 μg/mL significantly decreased the Oil Red O staining intensity of intestine and tail blood‐vessel fat in zebrafish (*p* < 0.01 at 0.195 μg/mL, *p* < 0.001 at 0.391 μg/mL; Figure 7b,d). For triptolide treatment, the staining intensity of intestine and tail blood‐vessel fat in zebrafish also decreased with increasing triptolide concentrations (*p* < 0.001 at 0.195 μg/mL; Figure 7i,k). These results indicate that both emodin and triptolide could dramatically inhibit fat absorption. In the NAD^+^/NADH assay, we found that emodin or triptolide treatment significantly increased the content of NAD^+^ (*p* < 0.001; Figure 7e,l). The content of NADH did not change significantly (Figure 7f,m), which eventually led to a significant increase in the NAD^+^/NADH ratio (*p* < 0.001; Figure 7g,n). The elevated NAD^+^/NADH ratio suggests that emodin and triptolide treatment could substantially enhance the metabolic rate of zebrafish.

**FIGURE 7:**
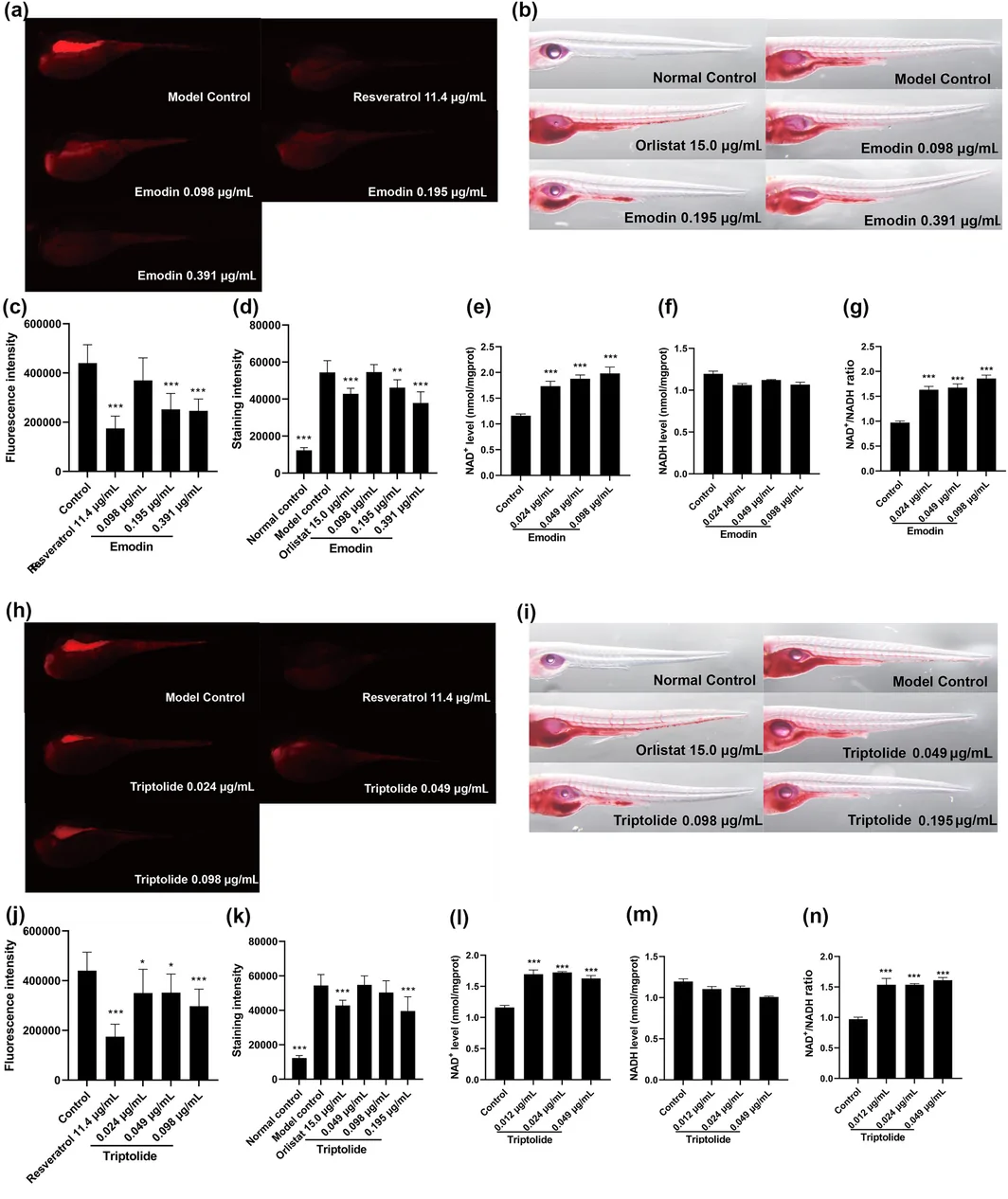
Emodin and triptolide promoted lipolysis, inhibited fat absorption, and upregulated the NAD^+^/NADH ratio in zebrafish. (a) Representative graph showing the fluorescent intensity of zebrafish yolk‐sac lipids after emodin treatment at concentrations of 0.098, 0.195, and 0.391 μg/mL. (b) Representative graph showing the lipid staining intensity in the zebrafish intestine and tail blood‐vessels after emodin treatment at concentrations of 0.098, 0.195, and 0.391 μg/mL. (c) Quantitative analysis of the fluorescence intensity of yolk‐sac lipids in zebrafish after emodin treatment. **p < 0.01 vs. the normal control group; ***p < 0.001 vs. the normal control group. (d) Quantitative analysis of the staining intensity of zebrafish intestinal and tail blood‐vessel fat after emodin treatment. **p < 0.01 vs. the model control group; ***p < 0.001 vs. the model control group. (e–g) NAD^+^ content (e), NADH content (f), and NAD^+^/NADH ratio (g) of zebrafish after emodin treatment at concentrations of 0.024, 0.049, and 0.098 μg/mL. ***p < 0.001 vs. the control group. (h) Representative graph showing the fluorescent intensity of zebrafish yolk‐sac lipids after triptolide treatment at concentrations of 0.024, 0.049, and 0.098 μg/mL. (i) Representative graph showing the lipid staining intensity in the zebrafish intestine and tail blood‐vessels after triptolide treatment at concentrations of 0.049, 0.098, and 0.195 μg/mL. (j) Quantitative analysis of the fluorescence intensity of yolk‐sac lipids in zebrafish after triptolide treatment. *p < 0.05 vs. the normal control group; ***p < 0.001 vs. the normal control group. (k) Quantitative analysis of the staining intensity of zebrafish intestinal and tail blood‐vessel fat after triptolide treatment. ***p < 0.001 vs. the model control group. (l–n) NAD^+^ content (l), NADH content (m), and NAD^+^/NADH ratio (n) of zebrafish after triptolide treatment at concentrations of 0.012, 0.024, and 0.049 μg/mL. ***p < 0.001 vs. the normal control group.

### 3.9 Emodin and Triptolide Regulate the AMPK Pathway in 3T3‐L1 Cells and Zebrafish

AMPK and SIRT1 are important molecules involved in the body's energy homeostasis. In 3T3‐L1 cells, emodin treatments significantly upregulated the SIRT1 protein expression (*p* < 0.05 at 5 and 10 μM, *p* < 0.01 at 25 μM, Figure 8a,b). Emodin also upregulated pAMPK protein expression, but a significant increase was only observed in the 25 μM treatment group (*p* < 0.05; Figure 8a,d). There were no significant differences in AMPK protein expression levels among the emodin treatment groups (Figure 8a,c). In zebrafish, similar to the positive control resveratrol, emodin treatment at concentrations of 0.098 μg/mL and 0.195 μg/mL significantly increased the mRNA expression levels of AMPK and SIRT1(Figure 8e,f).

**FIGURE 8:**
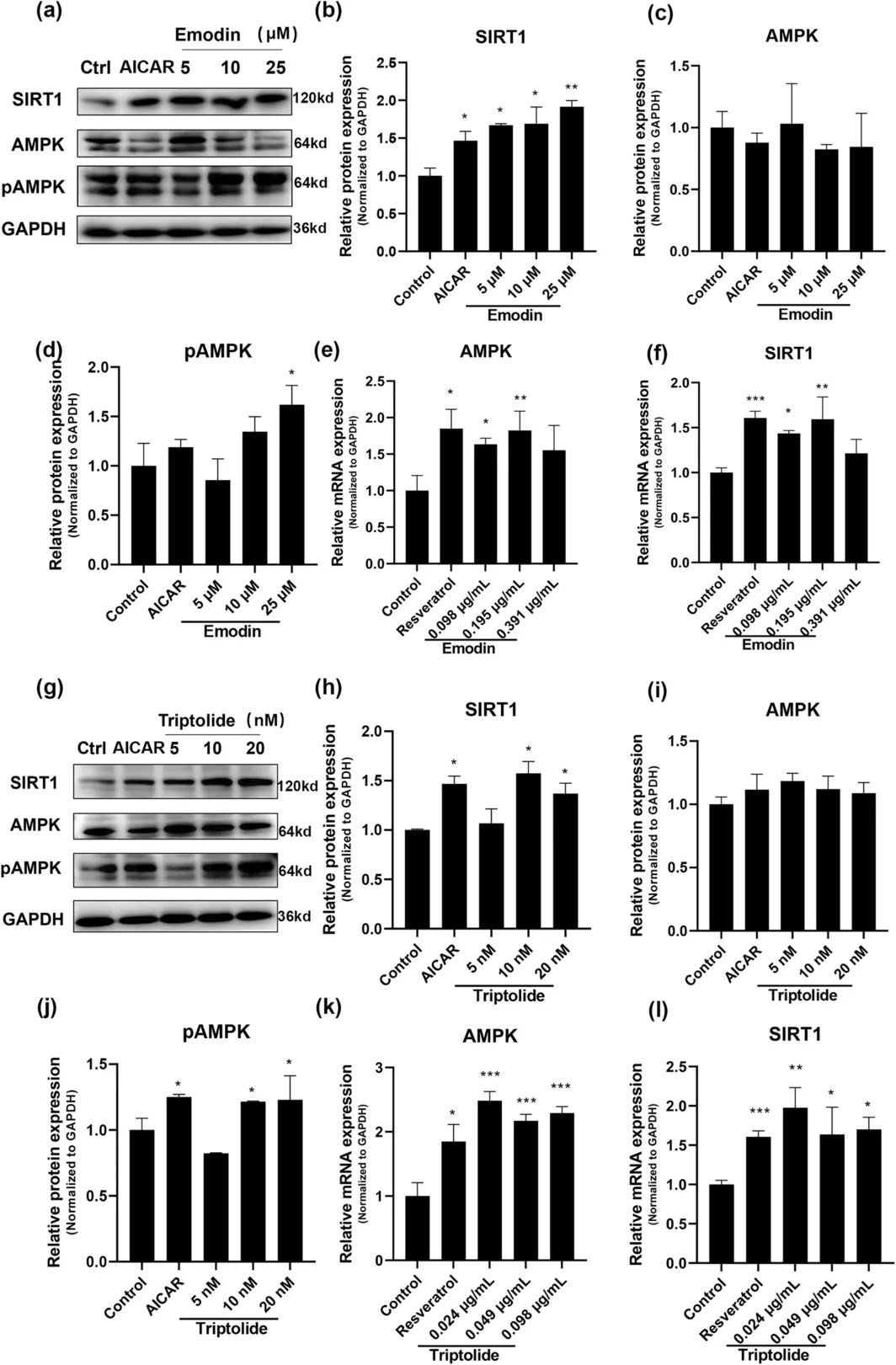
Emodin and triptolide regulated the AMPK/SIRT pathway in 3T3‐L1 cells and zebrafish. (a) Representative western blot image showing the expression levels of SIRT1, AMPK, and pAMPK in control and emodin‐treated 3T3‐L1 cells. (b–d) Quantification of SIRT1 (b), AMPK (c), and pAMPK (d) protein expression normalized to GAPDH in 3T3‐L1 cells. (e–f) qPCR analysis of AMPK (e) and SIRT1 (f) gene expression in control and emodin‐treated zebrafish. (g) Representative western blot images showing the expression levels of SIRT1, AMPK, and pAMPK in control and triptolide‐treated 3T3‐L1 cells. (h–j) Quantification of SIRT1 (h), AMPK (i), and pAMPK (j) protein expression normalized to GAPDH in 3T3‐L1 cells. (k–l) qPCR analysis of AMPK (k) and SIRT1 (l) gene expression in control and triptolide‐treated zebrafish. *p < 0.05 vs. the control group; **p < 0.01 vs. the control group; ***p < 0.001 vs. the control group.

In the 3T3‐L1 cells treated with triptolide, both 10 and 20 nM triptolide significantly upregulated the protein expression levels of SIRT1 and pAMPK (*p* < 0.05; Figure 8g,h,j). However, there were no significant differences in AMPK protein expression levels among triptolide treatment groups (Figure 8g,i). In zebrafish, triptolide treatment at all tested concentrations significantly upregulated the mRNA expression levels of AMPK (*p* < 0.001; Figure 8k) and SIRT1 (*p* < 0.01 at 0.024 μg/mL, *p* < 0.05 at 0.049 and 0.098 μg/mL; Figure 8l).

### 3.10 Emodin and Triptolide Regulate the Expression Levels of Genes Associated With Lipid Metabolism and Thermogenesis Pathways in 3T3‐L1 Cells

Subsequently, we conducted qPCR (Figure 9a,c) and Western blot (Figure 9b,d) experiments in 3T3‐L1 cells to investigate whether emodin and triptolide regulate the mRNA and protein expression levels of genes related to lipid metabolism and thermogenesis. For emodin, compared with the control group, emodin treatment upregulated the mRNA levels of thermogenesis‐associated genes. These included *PPARγ* (*p* < 0.05 at 5 μM; *p* < 0.01 at 10 μM; *p* < 0.001 at 25 μM), *C/EBPα* (*p* < 0.001 at 10 and 25 μM), *C/EBPβ* (*p* < 0.05 at 5 and 10 μM), *UCP1* (*p* < 0.05 at 5 μM), *PGC1α* (*p* < 0.001 at 10 μM; *p* < 0.01 at 25 μM), and *PRDM16* (*p* < 0.001 at 10 and 25 μM). It also upregulated the mRNA levels of mitochondrial biogenesis‐related genes such as *NRF1* (*p* < 0.001 at 10 μM and 25 μM), *NRF2* (*p* < 0.001 at 5 and 10 μM; *p* < 0.01 at 25 μM), and *TFAM* (*p* < 0.01 at 10 μM; *p* < 0.001 at 25 μM), β‐oxidation‐related genes *CPT1α* (*p* < 0.001 at 5, 10 and 25 μM) and *CPT1β* (*p* < 0.01 at 10 μM and 25 μM), as well as lipolysis‐related gene *HSL* (*p* < 0.001 at 10 and 25 μM). In contrast, emodin downregulated the mRNA levels of lipogenesis‐related genes *SREBP1* (*p* < 0.01 at 5 μM; *p* < 0.001 at 10 and 25 μM) and *ACC* (*p* < 0.05 at 10 μM; Figure 9a). Additionally, compared with the control group, emodin downregulated the protein levels of FASN (*p* < 0.05 at 25 μM) and pACC (*p* < 0.05 at 25 μM). Meanwhile, it upregulated the expression of thermogenesis‐related proteins including CPT1 (*p* < 0.05), UCP1 (*p* < 0.05 at 10 μM), PGC1α (*p* < 0.05 at 10 and 25 μM), C/EBPα (*p* < 0.05 at 10 and 25 μM), PPARγ (*p* < 0.05 at 10 and 25 μM), and PRDM16 (*p* < 0.05 at 25 μM; Figure 9b).

**FIGURE 9:**
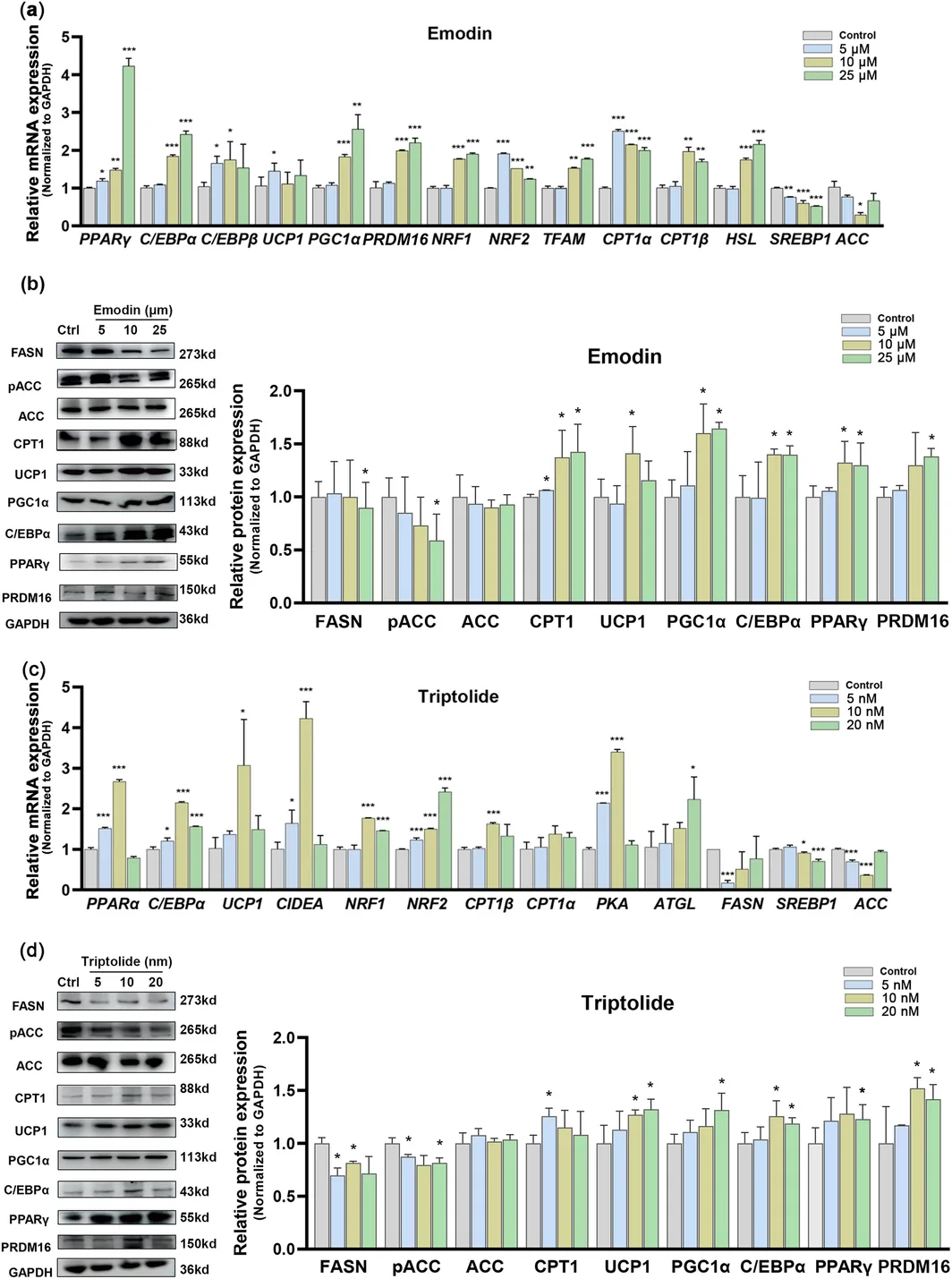
Emodin and triptolide regulated the expression levels of genes and proteins associated with thermogenesis, mitochondrial biogenesis, β‐oxidation, lipolysis, and lipogenesis in 3T3‐L1 cells. (a) qPCR analysis of genes involved in metabolic pathways in control and emodin‐treated 3T3‐L1 cells. (b) Representative western blot images and quantification of proteins involved in metabolic pathways in control and emodin‐treated 3T3‐L1 cells. (c) qPCR analysis of genes involved in metabolic pathways in control and triptolide‐treated 3T3‐L1 cells. (d) Representative western blot images and quantification of proteins involved in metabolic pathways in control and triptolide‐treated 3T3‐L1 cells. *p < 0.05 vs. the control group; **p < 0.01 vs. the control group; ***p < 0.001 vs. the control group.

Similarly, compared with the control group, triptolide upregulated the mRNA levels of thermogenesis‐related genes, including *PPARα* (*p* < 0.001 at 5 and 10 nM), *C/EBPα* (*p* < 0.05 at 5 nM; *p* < 0.001 at 10 and 20 nM), *UCP1* (*p* < 0.05 at 10 nM), and *CIDEA* (*p* < 0.05 at 5 nM; *p* < 0.001 at 10 nM). It also increased the mRNA levels of mitochondrial biogenesis‐related genes such as *NRF1* (*p* < 0.001 at 10 and 20 nM) and *NRF2* (*p* < 0.001). Moreover, triptolide upregulated the mRNA levels of β‐oxidation and lipolysis‐related genes, including *CPT1α*, *CPT1β* (*p* < 0.001 at 10 nM), *PKA* (*p* < 0.001 at 5 and 10 nM), and *ATGL* (*p* < 0.05 at 20 nM). In contrast, triptolide downregulated lipogenesis‐related genes such as *FASN* (*p* < 0.001 at 5 nM), *SREBP1* (*p* < 0.05 at 10 nM; *p* < 0.001 at 20 nM), and *ACC* (*p* < 0.001 at 5 and 10 nM; Figure 9c). Compared with the control group, triptolide downregulated the protein levels of FASN (*p* < 0.05 at 5 and 10 nM) and pACC (*p* < 0.05 at 5 and 20 nM). Meanwhile, it upregulated the protein levels of thermogenesis‐related molecules, including CPT1 (*p* < 0.05 at 5 nM), UCP1 (*p* < 0.05 at 10 and 20 nM), PGC1α (*p* < 0.05 at 20 nM), C/EBPα (*p* < 0.05 at 10 and 20 nM), PPARγ (*p* < 0.05 at 20 nM), and PRDM16 (*p* < 0.05 at 10 and 20 nM; Figure 9d).

## 4 Discussion

A deeper understanding of how excess body fat negatively impacts the factors contributing to T2DM pathogenesis can lead to innovative therapeutic interventions for preventing and treating T2DM (Klein et al. 2022). Exploring alternative weight loss agents, particularly natural products that stimulate adipose thermogenesis, offers exciting possibilities for the prevention and treatment of obesity (Fu et al. 2016). In TCM theory, the concept of the spleen governing transportation and metabolism of water and nutrients, along with Yin‐Yang principles, suggests that the spleen's function influences fat metabolism (Chung et al. 2016; Z. Liu 2009; Wu et al. 2024). When the spleen is weak, WAT tends to accumulate, leading to inefficient heat dissipation and the development of conditions such as obesity. Conversely, a strong spleen may promote the transformation of WAT into BAT, thereby enhancing energy expenditure and potentially preventing metabolic disorders. Therefore, through a review of English literature and the collection of TCMs that tonify the spleen and boost qi, 36 potential herbs have been identified that may promote adipose thermogenesis. Through the transcriptomic data analysis, we found TCM Da Huang may play a significant role in combating obesity through its thermogenesis‐inducing properties.

TCM Da Huang is well‐documented for its purgative, diuretic, heat‐clearing, blood‐cooling, and detoxifying properties, as per the Chinese Pharmacopoeia. Recent research has further illuminated its potential in regulating lipid metabolism and mitigating metabolic disorders (Cheng, Zhang, et al. 2021; Lee et al. 2019). Da Huang extract effectively lowers the blood lipids, TC, and LDL‐C in dyslipidemic rats and HepG2 cells, highlighting its therapeutic potential in managing lipid‐related disorders. The presence of anthraquinones such as rhein and emodin in Da Huang adds to its therapeutic repertoire. Emodin, another important anthraquinone, has demonstrated efficacy in improving glucose and lipid metabolism in high‐fat diet mice, and the mechanism is associated with the SREBP pathway (Li et al. 2016). Additionally, emodin's ability to activate BAT and promote the browning of WAT suggests its role in enhancing metabolic rate and energy expenditure (Cheng, Zhang, et al. 2021). Our network pharmacology analysis of Da Huang revealed that emodin possesses favorable drug properties and is predicted to bind to PPARγ, as indicated by molecular docking analysis. This led us to explore the thermogenesis‐enhancing effects of emodin in 3T3‐L1 adipocytes and zebrafish. Our experimental results suggested that emodin could upregulate genes associated with lipolysis, thermogenesis, β‐oxidation, and increase the NAD^+^/NADH ratio in 3T3‐L1 cells. Additionally, the zebrafish experiments showed that emodin could promote lipolysis, increase the NAD^+^/NADH ratio, and inhibit fat absorption. These findings collectively suggest that emodin, a major component of TCM Da Huang, holds promise as a therapeutic agent for metabolic disorders, particularly obesity‐related diseases.

CMap analysis to identify drug candidates is a powerful approach, as it leverages existing pharmacological data to predict new uses for known compounds. This accelerates the drug discovery process and reduces the risk associated with developing entirely new drugs. By inputting the thermogenesis signature genes into CMap analysis, we finally obtained lists of drugs showing similar expression patterns to the browning adipocyte phenotype. Triptolide was selected as the final candidate, and it is one of the active compounds of *T. wilfordii* Hook F. Celastrol, another compound from the same plant, has already demonstrated obesity‐treating properties (Liu et al. 2015; Ma et al. 2015; Wang et al. 2014). Liu et al. used CMap drug screening methods and first identified celastrol as a leptin sensitizer that can ameliorate obesity in high‐fat diet‐induced obese mice (Liu et al. 2015). Triptolide's previously established roles in anticancer and anti‐inflammatory effects suggest a multifaceted pharmacological profile, which might explain its broad applicability. However, its beneficial metabolic effects are rarely studied (Tong et al. 2021). Triptolide's potential lies in its ability to upregulate key metabolic pathways such as AMPK activation and lipolysis, as evidenced by its effects on P53 and ATGL transcription (Huang et al. 2021; Wang, Xu, et al. 2020). Therefore, our study aimed to investigate the thermogenesis‐upregulating effects of triptolide in 3T3‐L1 adipocytes and zebrafish. Our experimental results demonstrated that triptolide could upregulate genes associated with lipolysis, thermogenesis, β‐oxidation, and increase the NAD^+^/NADH ratio in 3T3‐L1 cells. Additionally, the zebrafish experiments showed that triptolide could promote lipolysis, increase the NAD^+^/NADH ratio, and inhibit fat absorption. PAR2 is a G‐protein coupled receptor involved in inflammation and has been linked to the development of diet‐induced obesity. Activation of PAR2 occurs through the cleavage of its N‐terminal domain by tissue factor and other serine proteases, a process that can result in decreased AMPK activity (Kim et al. 2021; Reynolds and Ives 2022). Our study discovered that triptolide is predicted to bind to PAR2. Through qPCR experiment, we found that triptolide downregulated the expression of PAR2 in zebrafish (data not shown) and upregulated AMPK. While triptolide shows great promise in promoting thermogenesis and lipolysis, further studies are needed to fully understand triptolide‐PAR2 interaction and to explore the safety and long‐term efficacy of using triptolide as a therapeutic agent for obesity and related metabolic disorders.

The AMPK/SIRT1 pathway is a critical regulator of cellular energy metabolism, and its dysregulation is closely linked to metabolic disorders such as obesity and T2DM (Steinberg and Carling 2019). Compounds like berberine, ginsenosides, resveratrol, and curcumin have demonstrated significant efficacy in upregulating AMPK/SIRT1, offering a natural alternative to synthetic drugs. AMPK stimulates thermogenesis by activating genes involved in thermogenesis (Wu et al. 2018). UCP1, regulated by PPARγ and C/EBPβ, is a key protein in thermogenesis, dissipating the proton gradient to generate heat (Villarroya et al. 2017). PGC‐1α enhances the transcriptional activity of transcription factors such as C/EBPβ and PPARγ, increasing the expression of UCP1 and promoting thermogenesis in adipocytes. PGC‐1α induces the transcription of nuclear respiratory factors, such as NRF1 and NRF2, which in turn trigger the expression of TFAM and other nuclear‐encoded subunits of the mitochondrial respiratory chain complexes (Ruderman et al. 2010). AMPK and SIRT1 regulate fatty acid oxidation through phosphorylation or deacetylation, such as by enhancing the activity of CPT1α, CPT1β and PPARα (Majeed et al. 2021). Similar to AMPK, SIRT1 can regulate lipolysis by activating PKA, ATGL, and HSL (Kim et al. 2016). AMPK inhibits the activity of SREBP1 by phosphorylating its inhibitory factor, thereby suppressing lipogenesis. AMPK phosphorylates ACC, inhibiting its activity and reducing fatty acid synthesis (Göransson et al. 2023; Li et al. 2011). Based on our current results, we hypothesize that emodin and triptolide could upregulate AMPK/SIRT1 by increasing the NAD^+^/NADH ratio, thereby promoting the expression of thermogenesis‐related genes (Figure 10). These transcriptional coactivators, such as PGC‐1α and PRDM16, can enhance UCP1 expression. Moreover, upregulated PGC‐1α can promote the transcription of factors like NRF and TFAM, facilitating mitochondrial biogenesis and energy expenditure. The upregulated AMPK/SIRT1 pathway activates HSL to enhance triglyceride hydrolysis, which further activates CPT1α and CPT1β, promoting the β‐oxidation process. Activated AMPK/SIRT1 can also inhibit lipogenesis‐related proteins such as ACC, SREBP‐1c, and FASN through phosphorylation or deacetylation.

**FIGURE 10:**
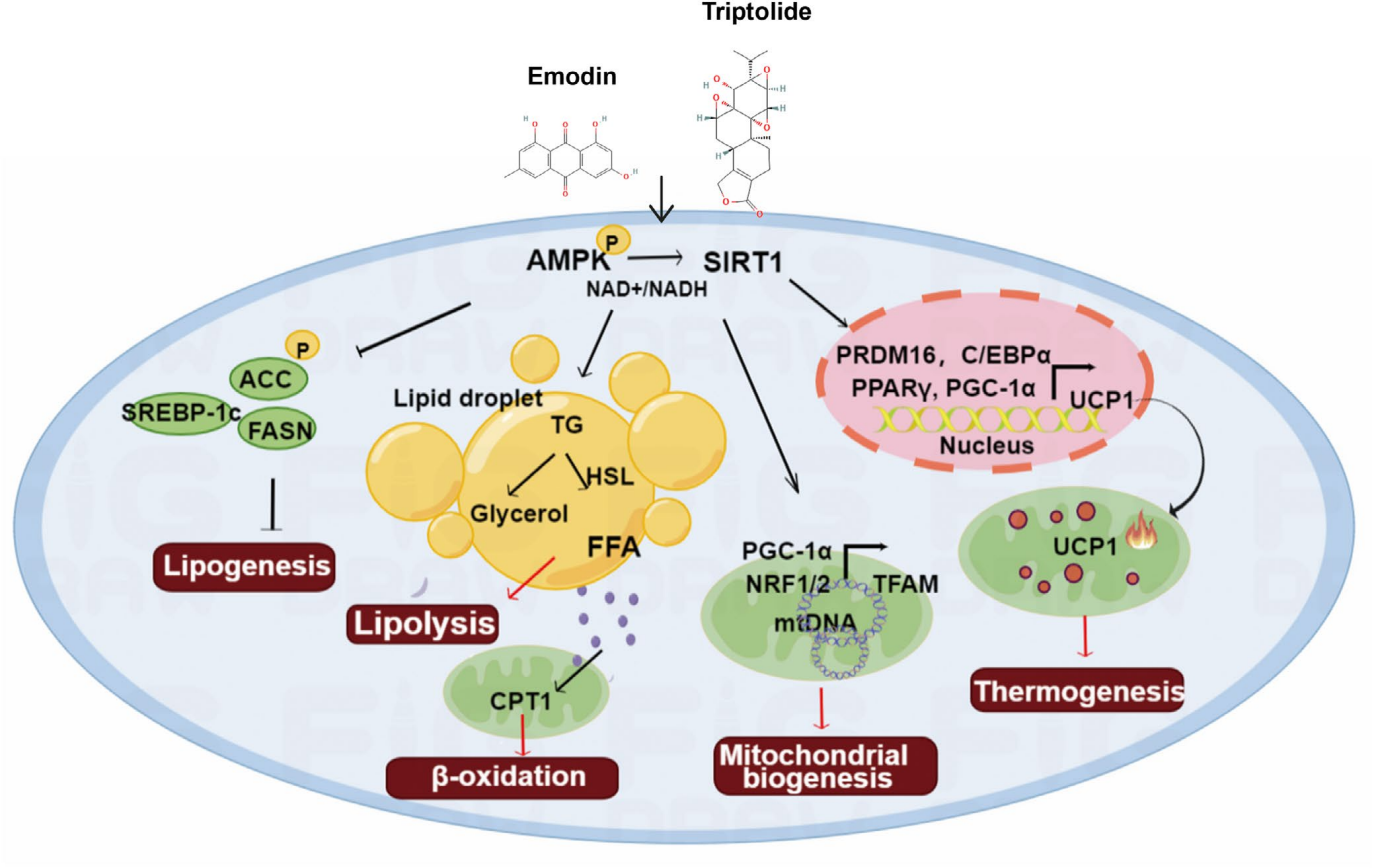
Mechanism of emodin and triptolide promoting energy expenditure in adipocytes.

## 5 Conclusion

In summary, we combined transcriptomic data analysis with network pharmacology, molecular docking, and CMap analysis to identify two potential drug candidates: emodin and triptolide, which activate adipocyte thermogenesis. The in vitro and in vivo experiments indicated that emodin and triptolide promoted thermogenesis and lipid metabolism‐related gene expression. Besides, they upregulated the NAD^+^/NADH ratio, as well as the important energy‐related pathway AMPK/SIRT1. Furthermore, our molecular docking analysis results suggested that emodin could bind to PPARγ, and triptolide could bind to PAR2. However, the direct interaction between emodin‐PPARγ and triptolide‐PAR2 still requires experimental validation, which will be the focus of our next research direction. By studying gene expression profiles in response to different TCM treatments, researchers can better understand the pathways and processes involved in the therapeutic effects of TCMs. This work could potentially lead to the development of novel therapeutics or treatments for obesity and T2DM. Ultimately, this research may yield new insights into the role of TCM in the prevention and management of T2DM from the energy expenditure perspective.

## Author Contributions

**Jing Li:** conceptualization, data curation, investigation, validation, visualization, writing – original draft, writing – review and editing. **Yang‐Chi‐Dung Lin:** project administration, supervision, writing – original draft. **Hsi‐Yuan Huang:** conceptualization, methodology, resources, writing – review and editing. **Jihang Chen:** resources, writing – review and editing. **Huali Zuo:** investigation, software. **Yun Tang:** investigation, validation. **Hsien‐Da Huang:** conceptualization, funding acquisition.

## Funding

This work was financially supported by the Shenzhen Science and Technology Program (JCYJ20250604141235046, JCYJ20250604141041017); Science and Technology Plan Project of Shenzhen (ZDCY20250901102000001); the Guangdong S&T Program (2024A0505050001, 2024A0505050002); the Warshel Institute for Computational Biology funding from Shenzhen City and Longgang District (LGKCSDPT2025001); the National Key R&D Program of China (2024YFE0104900) & Infinitus (China) Company Ltd. (2025E0155).

## Conflicts of Interest

The authors declare no conflicts of interest.

## Supporting information


**Figure S1:** Construction of mature adipocyte and zebrafish models. (a) Induction of 3T3‐L1 cell differentiation. (b) Construction of zebrafish model for lipolysis assay. (c) Construction of zebrafish model for fat absorption assay. (d) Construction of zebrafish model for NAD+/NADH assay.
**Figure S2:** 3T3‐L1 cell viability treated with different drugs assessed by the CCK‐8 assay.
**Figure S3:** Network of anthraquinones compounds from TCM Da Huang and their protein targets.

**Table S1:** Single herbs obtained from the literature and TCM theory, along with their treatment concentrations.
**Table S2:** Five datasets selected for the thermogenesis marker genes collection.
**Table S3:** Real‐time PCR primer sequences.
**Table S4:** Antibodies used in the study.
**Table S5:** Maximum testing concentration (MTC) for lipolysis assay in albino strain zebrafish.
**Table S6:** MTC for fat absorption assay in albino strain zebrafish.
**Table S7:** MTC for NAD^+^/NADH ratio assay in wild‐type AB strain zebrafish.

**Table S8:** Differentially expressed genes identified from RNA—sequencing of 3T3—L1 cells treated with TCMs.
**Table S9:** 137 compounds of TCM Da Huang collected from TCMs databases.
**Table S10:** Structural similarity analysis of TCM Da Huang.
**Table S11:** ADME analysis of compounds from TCM Da Huang.

## Data Availability

Data available on request from the authors.
