# Supplementary material for: Transcriptome Inference and Systematic Approaches to Investigate TCM Compounds With Beneficial Metabolic Effects
Source: Phytother Res. 2026 Jul 15;40(11):6154–72. doi: 10.1002/ptr.70407 (PMC13753672; doi:10.1002/ptr.70407)
Supplement: Supplementary file 1 [file PTR-40-6154-s002.docx]

**Supplementary figures:**

**Figure S1. Construction of Mature Adipocyte and Zebrafish Models. (a) Induction of 3T3-L1 Cell Differentiation. (b) Construction of Zebrafish Model for Lipolysis Assay. (c) Construction of Zebrafish Model for Fat Absorption Assay. (d) Construction of Zebrafish Model for NAD+/NADH Assay.**

**Figure S2.** **3T3-L1 cell viability treated with different drugs assessed by the CCK-8 assay**

**Figure S3. Network of anthraquinones compounds from TCM Da Huang and their protein targets.**
