# Supplementary material for: Transcriptome Inference and Systematic Approaches to Investigate TCM Compounds With Beneficial Metabolic Effects
Source: Phytother Res. 2026 Jul 15;40(11):6154–72. doi: 10.1002/ptr.70407 (PMC13753672; doi:10.1002/ptr.70407)
Supplement: Supplementary file 2 [file PTR-40-6154-s003.docx]

**Table S1. Single herbs obtained from the literature and TCM theory, along with their treatment concentrations.**

| **TCM** | Latin Name | **Lot #** |  | **L (g/ml)** | **M (g/ml)** | **H (g/ml)** |
| --- | --- | --- | --- | --- | --- | --- |
| Gan Cao | *Glycyrrhizae Radix Et Rhizoma* | 9056473 |  | 0.01414 | 0.009829 | 0.005507 |
| Gan Jiang | *Zingiberis Rhizoma* | 9061303 |  | 0.02486 | 0.015647 | 0.007483 |
| Da Huang | *Rhei Radix et Rhizoma* | 9065743 |  | 0.00709 | 0.005356 | 0.003426 |
| Huang Qin | *Scutellariae Radix* | 9066723 |  | 0.005152 | 0.003169 | 0.001462 |
| Dang Gui | *Angelicae Sinensis Radix* | 9076813 |  | 0.05044 | 0.040049 | 0.027732 |
| Zhi Shi | *Aurantii Fructus Immaturus* | 9060793 |  | 0.04239 | 0.035223 | 0.026223 |
| Hou Pu | *Magnoliae Officinalis Cortex* | 9086053 |  | 0.03566 | 0.033488 | 0.030296 |
| Huang Lian | *Coptidis Rhizoma* | 9035903 |  | 0.003277 | 0.002366 | 0.001408 |
| Zhi Zi | *Gardeniae Fructus* | 9071323 |  | 0.1 | 0.096505 | 0.091188 |
| Ze Xie | *Alismatis Rhizoma* | 9081933 |  | 0.09486 | 0.087828 | 0.077684 |
| Huang Qi | *Astragali Radix* | 9080293 |  | 0.09597 | 0.088799 | 0.078464 |
| Ge Gen | *Puerariae Lobatae Radix* | 9060033 |  | 0.01232 | 0.006749 | 0.002588 |
| Rou Gui | *Cinnamomi Cortex* | 9081243 |  | 0.02473 | 0.018668 | 0.011927 |
| Bai Zhi | *Angelicae Dahuricae Radix* | 9072003 |  | 0.08782 | 0.065289 | 0.040711 |
| Bo He | *Menthae Haplocalycis Herba* | 9065458 |  | 0.03098 | 0.029378 | 0.026996 |
| Chen Pi | *Citri Reticulatae Pericarpium* | 9051633 |  | 0.03597 | 0.033837 | 0.030697 |
| Dan Shen | *Salviae Miltiorrhizae Radix Et Rhizoma* | 9090193 |  | 0.004813 | 0.003696 | 0.002426 |
| He Ye | *Nelumbinis Folium* | 9061153 |  | 0.03619 | 0.033744 | 0.030185 |
| Ling Zhi | *Ganoderma* | 9096093 |  | 0.1083 | 0.101022 | 0.090424 |
| Shan Zha | *Crataegi Fructus* | 9032443 |  | 0.05072 | 0.042893 | 0.03284 |
| Gui Zhi | *Cinnamomi Ramulus* | 9080633 |  | 0.04113 | 0.038896 | 0.035584 |
| Ren Shen | *Ginseng Radix et Rhizoma* | 9085933 |  | 0.03083 | 0.028716 | 0.025644 |
| Fu Ling | *Poria* | 9101013 |  | 0.9157 | 0.860938 | 0.780375 |
| Bai Zhu | *Atractylodis Macrocephalae Rhizoma* | 9080693 |  | 0.09516 | 0.085348 | 0.071762 |
| Ma Huang | *Ephedrae Herba* | 9055653 |  | 0.02472 | 0.009643 | 0.002152 |
| Wu Zhu Yu | *Euodiae Fructus* | 9055983 |  | 0.01062 | 0.009954 | 0.008978 |
| Fa Ban Xia | *Pinelliae Rhizoma Praeparatum* | 9061793 |  | 0.3751 | 0.346047 | 0.304336 |
| Cang Zhu | *Atractylodis Rhizoma* | 9090712 |  | 0.03897 | 0.032637 | 0.024604 |
| Dang Shen | *Codonopsis Radix* | 9085553 |  | 0.05939 | 0.039793 | 0.021026 |
| Jue Ming Zi | *Cassiae Semen* | 9060233 |  | 0.1086 | 0.058773 | 0.022098 |
| Dou Kou | *Amomi Fructus Rotundus* | 9100803 |  | 0.138 | 0.12041 | 0.096898 |
| Lei Gong Teng | *Tripterygii Wilfordii Radix* | 9046253 |  | 0.02236 | 0.012101 | 0.00455 |
| Fo Shou | *Citri Sarcodactylis Fructus* | 9020193 |  | 0.1048 | 0.097268 | 0.08637 |
| Ji Xue Teng | *Spatholobi Caulis* | 9060403 |  | 0.0362 | 0.02755 | 0.017831 |
| Tu Fu Ling | *Smilacis Glabrae Rhizoma* | 9075843 |  | 0.03594 | 0.034613 | 0.0326 |
| Wu Mei | *Mume Fructus* | 9070063 |  | 0.02544 | 0.023539 | 0.020799 |

**Table S2.** **Five datasets selected for the thermogenesis marker genes collection.**

| **Series** | **Sample** | **Platform** |
| --- | --- | --- |
| GSE131861 | WAT:6; BAT:6 | GPL17021 Illumina HiSeq 2500 (Mus musculus) |
| GSE86338 | WAT:2, BAT:2 | GPL13112 Illumina HiSeq 2000 (Mus musculus) |
| GSE98132 | WAT:3, BAT:3 | GPL17021 Illumina HiSeq 2500 (Mus musculus) |
| GSE73266 | WAT:14, BAT:14 | GPL1261 [Mouse430_2] Affymetrix Mouse Genome 430 2.0 Array |
| GSE37248 | WAT:4; BAT:5 | GPL6246 [MoGene-1_0-st] Affymetrix Mouse Gene 1.0 ST Array [transcript (gene) version] |

Notes: WAT, white adipose tissue; BAT, brown adipose tissue.

**Table S3.** Real-time PCR primer sequences

| Gene Names | Forward (5’-3’) | Reverse (5’-3’) | Species |
| --- | --- | --- | --- |
| *PPARα* | ACCACTACGGAGTTCACGCATG | GAATCTTGCAGCTCCGATCACAC | Mus musculus |
| *PPARγ* | GTACTGTCGGTTTCAGAAGTGCC | ATCTCCGCCAACAGCTTCTCCT | Mus musculus |
| *C/EBPα* | GCAAAGCCAAGAAGTCGGTGGA | CCTTCTGTTGCGTCTCCACGTT | Mus musculus |
| *C/EBPβ* | CAACCTGGAGACGCAGCACAAG | GCTTGAACAAGTTCCGCAGGGT | Mus musculus |
| *UCP1* | GCTTTGCCTCACTCAGGATTGG | CCAATGAACACTGCCACACCTC | Mus musculus |
| *CIDEA* | GGTGGACACAGAGGAGTTCTTTC | CGAAGGTGACTCTGGCTATTCC | Mus musculus |
| *PGC1α* | GAATCAAGCCACTACAGACACCG | CATCCCTCTTGAGCCTTTCGTG | Mus musculus |
| *PRDM16* | ATCCACAGCACGGTGAAGCCAT | ACATCTGCCCACAGTCCTTGCA | Mus musculus |
| *NRF1* | GGCAACAGTAGCCACATTGGCT | GTCTGGATGGTCATTTCACCGC | Mus musculus |
| *NRF2* | CAGCATAGAGCAGGACATGGAG | GAACAGCGGTAGTATCAGCCAG | Mus musculus |
| *TFAM* | GAGGCAAAGGATGATTCGGCTC | CGAATCCTATCATCTTTAGCAAGC | Mus musculus |
| *CPT1α* | GGCATAAACGCAGAGCATTCCTG | CAGTGTCCATCCTCTGAGTAGC | Mus musculus |
| *CPT1β* | ATGTATCGCCGCAAACTGGACC | CTCTGAGAGGTGCTGTAGCAAG | Mus musculus |
| *ATGL* | GGAACCAAAGGACCTGATGACC | ACATCAGGCAGCCACTCCAACA | Mus musculus |
| *PKA* | GCTGGACTTCATTGGCTGTA | CATATTGCCGAACAGATTGG | Mus musculus |
| *HSL* | GCTCATCTCCTATGACCTACGG | TCCGTGGATGTGAACAACCAGG | Mus musculus |
| *SREBP1* | CGACTACATCCGCTTCTTGCAG | CCTCCATAGACACATCTGTGCC | Mus musculus |
| *FASN* | CACAGTGCTCAAAGGACATGCC | CACCAGGTGTAGTGCCTTCCTC | Mus musculus |
| *ACC* | GTTCTGTTGGACAACGCCTTCAC | GGAGTCACAGAAGCAGCCCATT | Mus musculus |
| *SIRT1* | GGAGCAGATTAGTAAGCGGCTTG | GTTACTGCCACAGGAACTAGAGG | Mus musculus |
| *GAPDH* | CATCACTGCCACCCAGAAGACTG | ATGCCAGTGAGCTTCCCGTTCAG | Mus musculus |
| *β-ACTIN* | TCGAGCAGGAGATGGGAACC | CTCGTGGATACCGCAAGATTC | Danio rerio |
| *AMPK* | AGCATGAGCTGACGAAGCA | TCCTTCTCATCCAACTTGCCA | Danio rerio |
| *SIRT1* | GGCTCCTGAACGAGAGGAAG | CGGGGTAGATTTCCTTGGCA | Danio rerio |
| *UCP1* | GGTGTGGGCAGACGATACAA | ACGGGAGATTGTCTGACAGG | Danio rerio |
| *PPARγ* | CTCCGCTGATATGGTGGACA | CGTGGAGAAGGGCTTGATGT | Danio rerio |
| *PAR2* | CTGCCCAGCCAGGCAAAAAT | ACTGCTCTGCAGTGTTTCTCT | Danio rerio |
| *β-ACTIN* | TCGAGCAGGAGATGGGAACC | CTCGTGGATACCGCAAGATTC | Homo sapiens |

**Table S4. Antibodies used in the study**

| **Species** | **Anti-body Names** | **Supplier** | **Dilution** | **Catalog Number** |
| --- | --- | --- | --- | --- |
| Rabbit | Anti-PPARγ | Cell Signaling Technology | 1/1000 | #2435T |
| Rabbit | Anti-AMPK | Sigma-Aldrich | 1/2000 | ab32047 |
| Rabbit | Anti-pAMPK | Sigma-Aldrich | 1/1000 | ab133448 |
| Rabbit | Anti-SirT1 | Cell Signaling Technology | 1/1000 | # 9475T |
| Rabbit | Anti-UCP1 | Sigma-Aldrich | 1/5000 | ab209483 |
| Rabbit | Anti-CEBP Alpha | Sigma-Aldrich | 1/1000 | ab40764 |
| Rabbit | Anti-PGC1 | Sigma-Aldrich | 1/1000 | ab176328 |
| Rabbit | Anti-CPT1a | Sigma-Aldrich | 1/1000 | ab234111 |
| Rabbit | Anti-ACC | Sigma-Aldrich | 1/1000 | ab45174 |
| Rabbit | Anti-pACC | Sigma-Aldrich | 1/5000 | ab68191 |
| Rabbit | Anti-PRDM16 | Sigma-Aldrich | 1/1000 | ab303534 |
| Rabbit | Anti-FASN | Sigma-Aldrich | 1/1000 | ab128856 |

**Table S5. Maximum testing concentration (MTC) for lipolysis assay in albino strain zebrafish.**

| **Group** | **Concentration (μg/mL)** | **Number of deaths** | **Mortality rate (%)** | **Phenotype** |
| --- | --- | --- | --- | --- |
| **Normal control** | / | 0 | 0 | No significant abnormality |
| **Emodin** | 0.195 | 0 | 0 | Similar to the normal control group |
|  | 0.391 | 0 | 0 | Similar to the normal control group |
|  | 0.781 | 0 | 0 | Severe compared with normal control group |
|  | 1.56 | 30 | 100 | / |
|  | 3.12 | 30 | 100 | / |
| **Triptolide** | 0.049 | 0 | 0 | Similar to the normal control group |
|  | 0.098 | 0 | 0 | Similar to the normal control group |
|  | 0.195 | 0 | 0 | Severe compared with normal control group |
|  | 0.391 | 0 | 0 | Severe compared with normal control group |
|  | 0.781 | 0 | 0 | Severe compared with normal control group |

**Table S6.** **MTC for fat absorption assay in albino strain zebrafish.**

| **Group** | **Concentration (μg/mL)** | **Number of deaths** | **Mortality rate (%)** | **Phenotype** |
| --- | --- | --- | --- | --- |
| **Normal control** | / | 0 | 0 | No significant abnormality |
| **Model control** | / | 0 | 0 | No significant abnormality |
| **Emodin** | 0.098 | 0 | 0 | Similar to model control group |
|  | 0.195 | 0 | 0 | Similar to model control group |
|  | 0.391 | 0 | 0 | Similar to model control group |
|  | 0.781 | 30 | 100 | / |
|  | 1.56 | 30 | 100 | / |
| **Triptolide** | 0.049 | 0 | 0 | Similar to the model control group |
|  | 0.098 | 0 | 0 | Similar to the model control group |
|  | 0.195 | 0 | 0 | Similar to the model control |
|  | 0.391 | 1 | 3 | Severe compared with model control group |
|  | 0.781 | 8 | 27 | / |

**Table S7.** **MTC for NAD^+^/NADH ratio assay in wild-type AB strain zebrafish**

| **Group** | **Concentration (μg/mL)** | **Number of deaths** | **Mortality rate (%)** | **Phenotype** |
| --- | --- | --- | --- | --- |
| **Normal control** | / | 0 | 0 | No significant abnormality |
| **Emodin** | 0.049 | 0 | 0 | Similar to normal control group |
|  | 0.098 | 0 | 0 | Similar to normal control group |
|  | 0.195 | 0 | 0 | Severe compared with normal control group |
|  | 0.391 | 18 | 60 | / |
|  | 0.781 | 30 | 100 | / |
| **Triptolide** | 0.024 | 0 | 0 | Similar to the normal control group |
|  | 0.049 | 0 | 0 | Similar to the normal control group |
|  | 0.098 | 4 | 13 | / |
|  | 0.195 | 16 | 53 | / |
|  | 0.391 | 30 | 100 | / |

**Table S8. Differentially expressed genes identified from RNA-sequencing of 3T3-L1 cells treated with TCMs**

**Table S9.** **137 compounds of TCM Da Huang collected from TCMs databases**.

| Compound | PubChem_id |
| --- | --- |
| Maleic acid | 444266 |
| Oxalic Acid | 971 |
| Citric Acid | 311 |
| D-malate | 92824 |
| Succinic Acid | 1110 |
| Gallic Acid | 740 |
| Tannic acid | 16129778 |
| beta-Glucogallin | 248042 |
| [(2R,3R,4S,5R,6R)-2,5-dihydroxy-6-(hydroxymethyl)-3-(3,4,5-trihydroxybenzoyl) oxy-tetrahydropyran-4-yl] 3,4,5-trihydroxybenzoate | 471118 |
| 6-O-Galloylglucose | 128839 |
| 2,3-Dihydroxypropyl 3,4,5-trihydroxybenzoate | 56861204 |
| 6-O-galloyl-beta-D-glucose | 5317463 |
| 1,6-bis-O-galloyl-beta-D-glucose | 440221 |
| alpha-D-Glucopyranose 1,3,6-tris(3,4,5-trihydroxybenzoate) | 11969003 |
| 1,2,6-Trigalloylglucose | 440308 |
| Gallic acid 3-O-(6'-O-galloyl)-beta-D-glucopyranoside | 11972353 |
| 3,5-dihydroxy-4-[(2R,3R,4S,5S,6R)-3,4,5-trihydroxy-6-[(3,4,5-trihydroxybenzoyl) oxymethyl] oxan-2-yl] oxybenzoic acid | 11968477 |
| 4-[(6-O-Galloyl-beta-D-glucopyranosyl) oxy]-4-deoxygallic acid | 11972354 |
| Piceatannol | 1335278 |
| Piceatannol 3'-O-glucoside | 11968990 |
| 5-(beta-D-glucopyranosyloxy)-2-hydroxybenzoic acid | 10914066 |
| Resveratroloside | 5322089 |
| (Z)-4'-(6-O-Galloyl-beta-D-glucopyranosyloxy) stilbene-3,5-diol | 11800639 |
| [(2R,3S,4S,5R,6S)-6-[4-[(E)-2-(3,5-dihydroxyphenyl) ethenyl] phenoxy]-3,4,5-trihydroxyoxan-2-yl] methyl 3,4,5-trihydroxybenzoate | 5322088 |
| Polydatin | 5281718 |
| Kaempferol | 5280863 |
| Cinnamic acid | 444539 |
| cis-Cinnamic acid | 5372954 |
| 1-O-trans-Cinnamoyl-beta-D-glucopyranose | 5280653 |
| [(2R,3R,4S,5S,6R)-2,4,5-trihydroxy-6-(hydroxymethyl) oxan-3-yl] (E)-3-phenylprop-2-enoate | 23944618 |
| 1-O-Galloyl-2-O-cinnamoyl-glucose | 5315898 |
| 1-O,6-O-Digalloyl-2-O-cinnamoyl-beta-D-glucopyranose | 6325082 |
| Cinchophen | 8593 |
| Rhein | 20336 |
| Aloe-emodin | 20414 |
| Aloe-emodin-glucoside | 294590 |
| Danthron | 2950 |
| Sennidin A | 92826 |
| Sennidin B | 10582718 |
| Sennidin C | 10642510 |
| Rheinoside A | 13888123 |
| (9R)-4,9-dihydroxy-10-oxo-9-[(2S,3R,4S,5S,6R)-3,4,5-trihydroxy-6-(hydroxymethyl) oxan-2-yl]-5-[(2S,3R,4S,5S,6R)-3,4,5-trihydroxy-6-(hydroxymethyl) oxan-2-yl] oxyanthracene-2-carboxylic acid | 13888124 |
| Rhein-8-glucoside | 5320961 |
| Aloe-emodin-8-O-beta-D-glucopyranoside | 5317644 |
| 9,10-dioxo-5-[(2S,3R,4S,5S,6R)-3,4,5-trihydroxy-6-(hydroxymethyl) oxan-2-yl] oxy-4-[(2S,3R,4S,5R,6R)-3,4,5-trihydroxy-6-(hydroxymethyl) oxan-2-yl] oxyanthracene-2-carboxylic acid | 5320960 |
| Rheinoside C | 13888128 |
| Sennoside A | 146222 |
| Sennoside B | 182880 |
| Sennoside | 656822 |
| Sennoside C | 46335769 |
| Sennoside D | 58995063 |
| Sennoside E | 73425506 |
| 4-hydroxy-9,10-dioxo-5-[(2S,3R,4S,5S,6R)-3,4,5-trihydroxy-6-(oxalooxymethyl) oxan-2-yl] oxyanthracene-2-carboxylic acid | 11972451 |
| Serotonin | 5202 |
| 3,5-Di-O-galloyl-4-O-digalloylquinic acid | 124022 |
| Procyanidin B6 | 474541 |
| (+)-Catechin 5-O-beta-D-glucopyranoside | 6324898 |
| 6-Glucopyranosylprocyanidin B1 | 21636073 |
| Procyanidin B1 | 11250133 |
| Procyanidin B4 | 147299 |
| Procyanidin B3 | 146798 |
| Procyanidin B2 | 122738 |
| procyanidin B1 3-O-gallate | 12795888 |
| 3,3'-Digalloylprocyanidin B2 | 124016 |
| (+)-Gallocatechin | 65084 |
| (+)-Epicatechin | 182232 |
| (-)-Catechin | 146320 |
| (-)-Epicatechin gallate | 107905 |
| Catechin 3'-O-gallate | 44257105 |
| 1-O-Galloylpedunculagin | 452242 |
| Benzylideneacetone | 637759 |
| (8S,9S,10R,13S,14R,17S)-17-acetyl-10,13-dimethyl-1,2,6,7,8,9,11,12,14,15,16,17-dodecahydrocyclopenta[a]phenanthren-3-one | 11876100 |
| Progesterone | 5994 |
| 4-(4-Hydroxyphenyl)-2-butanone | 21648 |
| Raspberryketone glucoside | 5320521 |
| CID 10390322 | 10390322 |
| Lindleyin | 42994 |
| Catechin pentaacetate | 5315742 |
| Epicatechin pentaacetate | 5317058 |
| D-Lactic acid | 61503 |
| CID 11799446 | 11799446 |
| Rottlerin | 5281847 |
| Tripterygone | 197388 |
| Citroxanthin | 5281246 |
| Laccaic acid D | 9883304 |
| 2-[(R)-2-Hydroxypropyl]-5-methyl-7-hydroxychromone | 1.02E+08 |
| 4H-1-Benzopyran-4-one, 5-acetyl-7-hydroxy-2-methyl- | 10631782 |
| 7-Hydroxy-2-methyl-4-oxo-4H-1-benzopyran-5-carboxylic acid | 10631376 |
| Cassiachromone | 5319500 |
| 7-Hydroxy-2-methyl-4-oxo-4H-1-benzopyran-5-acetic acid | 14429402 |
| 2-methyl-4-oxo-7-[(2S,3R,4S,5S,6R)-3,4,5-trihydroxy-6-(hydroxymethyl) oxan-2-yl] oxychromene-5-carboxylic acid | 5315687 |
| 2'-Methylacetophenone | 11340 |
| 1,8-Dihydroxy-3-methoxy-2,6-dimethylanthracene-9,10-dione | 21770756 |
| Emodin anthrone | 122635 |
| Chrysophanol-9-anthrone | 68111 |
| Emodin | 6440 |
| Physcion | 21278 |
| Emodin 6-O-\|A-D-glucoside | 5317038 |
| Emodin-8-glucoside | 99649 |
| Rheochrysin | 337876 |
| Physcion 8-gentiobioside | 885524 |
| Chrysophanol | 20416 |
| Pulmatin | 442731 |
| Chrysophanol 8-(6-O-galloyl-beta-D-glucopyranoside) | 5315852 |
| Palmidin C | 10640772 |
| Rheidin A | 5320957 |
| Rheidin C | 10641918 |
| Rheidin B | 10641916 |
| Palmidin A | 10640768 |
| Palmidin B | 10640770 |
| Emodin 1-O-beta-D-glucoside | 10638666 |
| Physcion 8-glucoside | 5319323 |
| Chrysophanein | 12649846 |
| 8-[(6-O-beta-D-Glucopyranosyl-beta-D-glucopyranosyl) oxy]-1-hydroxy-3-methoxy-6-methylanthraquinone | 5320543 |
| 1-[1-hydroxy-3-methyl-8-[(2S,3R,4S,5S,6R)-3,4,5-trihydroxy-6-(hydroxymethyl) oxan-2-yl] oxynaphthalen-2-yl] ethanone | 5319972 |
| 1-[1,6-dihydroxy-3-methyl-8-[(2S,3R,4S,5S,6R)-3,4,5-trihydroxy-6-(hydroxymethyl) oxan-2-yl] oxynaphthalen-2-yl] ethanone | 100753 |
| Torachrysone 8-O-Glucoside | 11972479 |
| Torachrysone 8-glucoside | 14345578 |
| Ethanone, 1-[8-[[6-O-(carboxycarbonyl)-beta-D-glucopyranosyl] oxy]-1-hydroxy-6-methoxy-3-methyl-2-naphthalenyl]- | 5321979 |
| 1-[1-hydroxy-6-methoxy-3-methyl-8-[(2S,3R,4S,5S,6R)-3,4,5-trihydroxy-6-[[(2R,3R,4S,5S,6R)-3,4,5-trihydroxy-6-(hydroxymethyl) tetrahydropyran-2-yl] oxymethyl] tetrahydropyran-2-yl]oxy-2-naphthyl]ethanone | 503732 |
| Toralactone | 5321980 |
| Afzelin | 5316673 |
| Phlinoside C | 6443993 |
| Phlinoside B | 6450096 |
| Phlinoside D | 6450124 |
| 2H-1-Benzopyran-5-acetic acid, 3,4-dihydro-7-hydroxy-2-methyl-4-oxo-, (S)- | 10639086 |
| Plantarenaloside | 155882 |
| 10beta-Hydroxy-6beta-isobutyrylfuranoeremophilane | 442377 |
| Beta-Sitosterol | 222284 |
| Sitogluside | 5742590 |
| 2-Methyl-5-(8Z,11Z)-8,11-pentadecadien-1-yl-1,3-benzenediol | 5319544 |
| Tripalmitin | 11147 |
| Docosanoic acid | 8215 |
| Rhapontigenin | 5320954 |
| 5-[(Z)-2-(3-hydroxy-4-methoxyphenyl) ethenyl] benzene-1,3-diol | 10879760 |
| Rhapontin | 1274426 |
| Eupatin | 5317287 |

**Table S10. Structural similarity analysis of TCM Da Huang**

| PUBCHEM ID | Compounds | Modules |
| --- | --- | --- |
| 370 | Gallic Acid | 1 |
| 5317465 | 2,3-Dihydroxypropyl 3,4,5-trihydroxybenzoate | 1 |
| 16129778 | Tannic acid | 1 |
| 124021 | beta-Glucogallin | 1 |
| 471118 | [(2R,3R,4S,5R,6R)-2,5-dihydroxy-6-(hydroxymethyl)-3-(3,4,5-trihydroxybenzoyl) oxy-tetrahydropyran-4-yl] 3,4,5-trihydroxybenzoate | 1 |
| 5317463 | 6-O-galloyl-beta-D-glucose | 1 |
| 440221 | 1,6-bis-O-galloyl-beta-D-glucose | 1 |
| 11969003 | alpha-D-Glucopyranose 1,3,6-tris(3,4,5-trihydroxybenzoate) | 1 |
| 440308 | 1,2,6-Trigalloylglucose | 1 |
| 11972353 | Gallic acid 3-O-(6'-O-galloyl)-beta-D-glucopyranoside | 1 |
| 11968477 | 3,5-dihydroxy-4-[(2R,3R,4S,5S,6R)-3,4,5-trihydroxy-6-[(3,4,5-trihydroxybenzoyl) oxymethyl] oxan-2-yl] oxybenzoic acid | 1 |
| 11972354 | 4-[(6-O-Galloyl-beta-D-glucopyranosyl) oxy]-4-deoxygallic acid | 1 |
| 11800639 | (Z)-4'-(6-O-Galloyl-beta-D-glucopyranosyloxy) stilbene-3,5-diol | 1 |
| 5322088 | [(2R,3S,4S,5R,6S)-6-[4-[(E)-2-(3,5-dihydroxyphenyl) ethenyl] phenoxy]-3,4,5-trihydroxyoxan-2-yl] methyl 3,4,5-trihydroxybenzoate | 1 |
| 5315898 | 1-O-Galloyl-2-O-cinnamoyl-glucose | 1 |
| 6325082 | 1-O,6-O-Digalloyl-2-O-cinnamoyl-beta-D-glucopyranose | 1 |
| 124022 | 3,5-Di-O-galloyl-4-O-digalloylquinic acid | 1 |
| 452242 | 1-O-Galloylpedunculagin | 1 |
| 10390322 | CID 10390322 | 1 |
| 42994 | Lindleyin | 1 |
| 128839 | 6-O-Galloylglucose | 1 |
| 11968990 | Piceatannol 3'-O-glucoside | 1 |
| 10914066 | 5-(beta-D-glucopyranosyloxy)-2-hydroxybenzoic acid | 1 |
| 5322089 | Resveratroloside | 1 |
| 5281718 | Polydatin | 1 |
| 5319972 | 1-[1-hydroxy-3-methyl-8-[(2S,3R,4S,5S,6R)-3,4,5-trihydroxy-6-(hydroxymethyl) oxan-2-yl] oxynaphthalen-2-yl] ethanone | 1 |
| 100753 | 1-[1,6-dihydroxy-3-methyl-8-[(2S,3R,4S,5S,6R)-3,4,5-trihydroxy-6-(hydroxymethyl) oxan-2-yl] oxynaphthalen-2-yl]ethanone | 1 |
| 11972479 | Torachrysone 8-O-Glucoside | 1 |
| 14345578 | Torachrysone 8-glucoside | 1 |
| 5321979 | Ethanone, 1-[8-[[6-O-(carboxycarbonyl)-beta-D-glucopyranosyl] oxy]-1-hydroxy-6-methoxy-3-methyl-2-naphthalenyl]- | 1 |
| 503732 | 1-[1-hydroxy-6-methoxy-3-methyl-8-[(2S,3R,4S,5S,6R)-3,4,5-trihydroxy-6-[[(2R,3R,4S,5S,6R)-3,4,5-trihydroxy-6-(hydroxymethyl) tetrahydropyran-2-yl] oxymethyl] tetrahydropyran-2-yl] oxy-2-naphthyl]ethanone | 1 |
| 637213 | Rhapontin | 1 |
| 5320521 | Raspberryketone glucoside | 1 |
| 667639 | Piceatannol | others |
| 5320954 | Rhapontigenin | others |
| 10879760 | 5-[(Z)-2-(3-hydroxy-4-methoxyphenyl) ethenyl] benzene-1,3-diol | others |
| 444539 | Cinnamic acid | others |
| 5372954 | cis-Cinnamic acid | others |
| 637759 | Benzylideneacetone | others |
| 5280653 | 1-O-trans-Cinnamoyl-beta-D-glucopyranose | others |
| 11972309 | [(2R,3R,4S,5S,6R)-2,4,5-trihydroxy-6-(hydroxymethyl) oxan-3-yl] (E)-3-phenylprop-2-enoate | others |
| 6443993 | Phlinoside C | others |
| 6450096 | Phlinoside B | others |
| 6450124 | Phlinoside D | others |
| 10168 | Rhein | 4 |
| 10207 | Aloe-emodin | 4 |
| 2950 | Danthron | 4 |
| 92826 | Sennidin A | 4 |
| 10459879 | Sennidin B | 4 |
| 5321255 | Sennidin C | 4 |
| 9883304 | Laccaic acid D | 4 |
| 21770756 | 1,8-Dihydroxy-3-methoxy-2,6-dimethylanthracene-9,10-dione | 4 |
| 122635 | Emodin anthrone | 4 |
| 68111 | Chrysophanol-9-anthrone | 4 |
| 3220 | Emodin | 4 |
| 10639 | Physcion | 4 |
| 10208 | Chrysophanol | 4 |
| 5320386 | Palmidin C | 4 |
| 5320957 | Rheidin A | 4 |
| 5320959 | Rheidin C | 4 |
| 5320958 | Rheidin B | 4 |
| 5320384 | Palmidin A | 4 |
| 5320385 | Palmidin B | 4 |
| 11972451 | 4-hydroxy-9,10-dioxo-5-[(2S,3R,4S,5S,6R)-3,4,5-trihydroxy-6-(oxalooxymethyl) oxan-2-yl] oxyanthracene-2-carboxylic acid | 2 |
| 5317038 | Emodin 6-O-\|A-D-glucoside | 2 |
| 5315852 | Chrysophanol 8-(6-O-galloyl-beta-D-glucopyranoside) | 2 |
| 5320961 | Rhein-8-glucoside | 2 |
| 5317644 | Aloe-emodin-8-O-beta-D-glucopyranoside | 2 |
| 5320960 | 9,10-dioxo-5-[(2S,3R,4S,5S,6R)-3,4,5-trihydroxy-6-(hydroxymethyl) oxan-2-yl] oxy-4-[(2S,3R,4S,5R,6R)-3,4,5-trihydroxy-6-(hydroxymethyl) oxan-2-yl] oxyanthracene-2-carboxylic acid | 2 |
| 442731 | Pulmatin | 2 |
| 6324923 | Chrysophanein | 2 |
| 294590 | Aloe-emodin-glucoside | 2 |
| 73111 | Sennoside A | 2 |
| 91440 | Sennoside B | 2 |
| 656822 | Sennoside | 2 |
| 46173829 | Sennoside C | 2 |
| 46173830 | Sennoside D | 2 |
| 73425506 | Sennoside E | 2 |
| 99649 | Emodin-8-glucoside | 2 |
| 168938 | Rheochrysin | 2 |
| 442762 | Physcion 8-gentiobioside | 2 |
| 5319333 | Emodin 1-O-beta-D-glucoside | 2 |
| 5319323 | Physcion 8-glucoside | 2 |
| 5320543 | 8-[(6-O-beta-D-Glucopyranosyl-beta-D-glucopyranosyl) oxy]-1-hydroxy-3-methoxy-6-methylanthraquinone | 2 |
| 13888123 | Rheinoside A | 2 |
| 13888124 | (9R)-4,9-dihydroxy-10-oxo-9-[(2S,3R,4S,5S,6R)-3,4,5-trihydroxy-6-(hydroxymethyl) oxan-2-yl]-5-[(2S,3R,4S,5S,6R)-3,4,5-trihydroxy-6-(hydroxymethyl) oxan-2-yl] oxyanthracene-2-carboxylic acid | 2 |
| 13888128 | Rheinoside C | 2 |
| 21636073 | 6-Glucopyranosylprocyanidin B1 | 3 |
| 12795888 | procyanidin B1 3-O-gallate | 3 |
| 124016 | 3,3'-Digalloylprocyanidin B2 | 3 |
| 474541 | Procyanidin B6 | 3 |
| 6324898 | (+)-Catechin 5-O-beta-D-glucopyranoside | 3 |
| 11250133 | Procyanidin B1 | 3 |
| 147299 | Procyanidin B4 | 3 |
| 146798 | Procyanidin B3 | 3 |
| 122738 | Procyanidin B2 | 3 |
| 65084 | (+)-Gallocatechin | 3 |
| 182232 | (+)-Epicatechin | 3 |
| 73160 | (-)-Catechin | 3 |
| 107905 | (-)-Epicatechin gallate | 3 |
| 44257105 | Catechin 3'-O-gallate | 3 |
| 5315742 | Catechin pentaacetate | 3 |
| 5317058 | Epicatechin pentaacetate | 3 |
| 11876100 | (8S,9S,10R,13S,14R,17S)-17-acetyl-10,13-dimethyl-1,2,6,7,8,9,11,12,14,15,16,17-dodecahydrocyclopenta[a]phenanthren-3-one | others |
| 5994 | Progesterone | others |
| 5315687 | 2-methyl-4-oxo-7-[(2S,3R,4S,5S,6R)-3,4,5-trihydroxy-6-(hydroxymethyl) oxan-2-yl] oxychromene-5-carboxylic acid | others |
| 5280863 | Kaempferol | others |
| 5316673 | Afzelin | others |
| 5317287 | Eupatin | others |
| 1.02E+08 | 2-[(R)-2-Hydroxypropyl]-5-methyl-7-hydroxychromone | others |
| 5315891 | 4H-1-Benzopyran-4-one, 5-acetyl-7-hydroxy-2-methyl- | others |
| 5315688 | 7-Hydroxy-2-methyl-4-oxo-4H-1-benzopyran-5-carboxylic acid | others |
| 5319500 | Cassiachromone | others |

**Table S11.** **ADME analysis of compounds from TCM Da Huang**

| Elements | Formula | MW | #Rotatable bonds | #H-bond acceptors | #H-bond donors | iLOGP | GI absorption | Lipinski #violations | Ghose #violations | Veber #violations | Egan #violations | Muegge #violations |
| --- | --- | --- | --- | --- | --- | --- | --- | --- | --- | --- | --- | --- |
| Maleic acid | C4H4O4 | 116.07 | 2 | 4 | 2 | 0.32 | High | 0 | 3 | 0 | 0 | 2 |
| Oxalic Acid | C2H2O4 | 90.03 | 1 | 4 | 2 | -0.35 | High | 0 | 4 | 0 | 0 | 2 |
| D-malate | C4H6O5 | 134.09 | 3 | 5 | 3 | -0.01 | High | 0 | 4 | 0 | 0 | 2 |
| Succinic Acid | C4H6O4 | 118.09 | 3 | 4 | 2 | 0.32 | High | 0 | 3 | 0 | 0 | 2 |
| Gallic Acid | C7H6O5 | 170.12 | 1 | 5 | 4 | 0.21 | High | 0 | 2 | 0 | 0 | 1 |
| Piceatannol | C14H12O4 | 244.24 | 2 | 4 | 4 | 1.61 | High | 0 | 0 | 0 | 0 | 0 |
| Kaempferol | C15H10O6 | 286.24 | 1 | 6 | 4 | 1.7 | High | 0 | 0 | 0 | 0 | 0 |
| Cinnamic acid | C9H8O2 | 148.16 | 2 | 2 | 1 | 1.55 | High | 0 | 2 | 0 | 0 | 1 |
| cis-Cinnamic acid | C9H8O2 | 148.16 | 2 | 2 | 1 | 1.55 | High | 0 | 2 | 0 | 0 | 1 |
| 1-O-trans-Cinnamoyl-beta-D-glucopyranose | C15H18O7 | 310.3 | 5 | 7 | 4 | 2.21 | High | 0 | 1 | 0 | 0 | 0 |
| [(2R,3R,4S,5S,6R)-2,4,5-trihydroxy-6-(hydroxymethyl) oxan-3-yl] (E)-3-phenylprop-2-enoate | C15H18O7 | 310.3 | 5 | 7 | 4 | 1.91 | High | 0 | 1 | 0 | 0 | 0 |
| Cinchophen | C16H11NO2 | 249.26 | 2 | 3 | 1 | 1.98 | High | 0 | 0 | 0 | 0 | 0 |
| Rhein | C15H8O6 | 284.22 | 1 | 6 | 3 | 1.28 | High | 0 | 0 | 0 | 0 | 0 |
| Aloe-emodin | C15H10O5 | 270.24 | 1 | 5 | 3 | 1.96 | High | 0 | 0 | 0 | 0 | 0 |
| Danthron | C14H8O4 | 240.21 | 0 | 4 | 2 | 1.96 | High | 0 | 0 | 0 | 0 | 0 |
| Serotonin | C10H12N2O | 176.22 | 2 | 2 | 3 | 1.18 | High | 0 | 0 | 0 | 0 | 1 |
| Benzylideneacetone | C10H10O | 146.19 | 2 | 1 | 0 | 2 | High | 0 | 1 | 0 | 0 | 2 |
| (8S,9S,10R,13S,14R,17S)-17-acetyl-10,13-dimethyl-1,2,6,7,8,9,11,12,14,15,16,17-dodecahydrocyclopenta[a]phenanthren-3-one | C21H30O2 | 314.46 | 1 | 2 | 0 | 3.08 | High | 0 | 0 | 0 | 0 | 0 |
| Progesterone | C21H30O2 | 314.46 | 1 | 2 | 0 | 3.08 | High | 0 | 0 | 0 | 0 | 0 |
| 4-(4-Hydroxyphenyl)-2-butanone | C10H12O2 | 164.2 | 3 | 2 | 1 | 1.72 | High | 0 | 0 | 0 | 0 | 1 |
| Raspberryketone glucoside | C16H22O7 | 326.34 | 6 | 7 | 4 | 1.67 | High | 0 | 1 | 0 | 0 | 0 |
| D-Lactic acid | C3H6O3 | 90.08 | 1 | 3 | 2 | 0.52 | High | 0 | 4 | 0 | 0 | 2 |
| Laccaic acid D | C16H10O7 | 314.25 | 1 | 7 | 4 | 0.95 | High | 0 | 0 | 0 | 1 | 0 |
| 2-[(R)-2-Hydroxypropyl]-5-methyl-7-hydroxychromone | C13H14O4 | 234.25 | 2 | 4 | 2 | 2.08 | High | 0 | 0 | 0 | 0 | 0 |
| 4H-1-Benzopyran-4-one, 5-acetyl-7-hydroxy-2-methyl- | C12H10O4 | 218.21 | 1 | 4 | 1 | 1.44 | High | 0 | 0 | 0 | 0 | 0 |
| 7-Hydroxy-2-methyl-4-oxo-4H-1-benzopyran-5-carboxylic acid | C11H8O5 | 220.18 | 1 | 5 | 2 | 1.19 | High | 0 | 0 | 0 | 0 | 0 |
| Cassiachromone | C13H12O4 | 232.23 | 2 | 4 | 1 | 1.86 | High | 0 | 0 | 0 | 0 | 0 |
| 7-Hydroxy-2-methyl-4-oxo-4H-1-benzopyran-5-acetic acid | C12H10O5 | 234.2 | 2 | 5 | 2 | 1.39 | High | 0 | 0 | 0 | 0 | 0 |
| 2'-Methylacetophenone | C9H10O | 134.18 | 1 | 1 | 0 | 1.87 | High | 0 | 1 | 0 | 0 | 2 |
| 1,8-Dihydroxy-3-methoxy-2,6-dimethylanthracene-9,10-dione | C17H14O5 | 298.29 | 1 | 5 | 2 | 2.71 | High | 0 | 0 | 0 | 0 | 0 |
| Emodin anthrone | C15H12O4 | 256.25 | 0 | 4 | 3 | 2.26 | High | 0 | 0 | 0 | 0 | 0 |
| Chrysophanol-9-anthrone | C15H12O3 | 240.25 | 0 | 3 | 2 | 2.56 | High | 0 | 0 | 0 | 0 | 0 |
| Emodin | C15H10O5 | 270.24 | 0 | 5 | 3 | 1.8 | High | 0 | 0 | 0 | 0 | 0 |
| Physcion | C16H12O5 | 284.26 | 1 | 5 | 2 | 2.45 | High | 0 | 0 | 0 | 0 | 0 |
| Chrysophanol | C15H10O4 | 254.24 | 0 | 4 | 2 | 2.22 | High | 0 | 0 | 0 | 0 | 0 |
| Toralactone | C15H12O5 | 272.25 | 1 | 5 | 2 | 2.79 | High | 0 | 0 | 0 | 0 | 0 |
| 2H-1-Benzopyran-5-acetic acid, 3,4-dihydro-7-hydroxy-2-methyl-4-oxo-, (S)- | C12H12O5 | 236.22 | 2 | 5 | 2 | 0.69 | High | 0 | 0 | 0 | 0 | 0 |
| 10beta-Hydroxy-6beta-isobutyrylfuranoeremophilane | C19H28O4 | 320.42 | 3 | 4 | 1 | 3.48 | High | 0 | 0 | 0 | 0 | 0 |
| Rhapontigenin | C15H14O4 | 258.27 | 3 | 4 | 3 | 2.21 | High | 0 | 0 | 0 | 0 | 0 |
| 5-[(Z)-2-(3-hydroxy-4-methoxyphenyl)ethenyl] benzene-1,3-diol | C15H14O4 | 258.27 | 3 | 4 | 3 | 2.21 | High | 0 | 0 | 0 | 0 | 0 |

Notes: MW, molecular weight; GI: gastrointestinal.
